# CDK5 promotes apoptosis and attenuates chemoresistance in gastric cancer via E2F1 signaling

**DOI:** 10.1186/s12935-023-03112-4

**Published:** 2023-11-21

**Authors:** Long-Long Cao, Yu-Kai Wu, Tong-Xin Lin, Mi Lin, Yu-Jing Chen, Ling-Qian Wang, Jia-Bin Wang, Jian-Xian Lin, Jun Lu, Qi-Yue Chen, Ru-Hong Tu, Ze-Ning Huang, Ju-Li Lin, Hua-Long Zheng, Jian-Wei Xie, Ping Li, Chang-Ming Huang, Chao-Hui Zheng

**Affiliations:** 1 Department of Gastric Surgery, Fujian Medical University Union Hospital, No.29 Xinquan Road, Fuzhou, Fujian Province 350001 China; 2 Key Laboratory of Ministry of Education of Gastrointestinal Cancer, Fujian Medical University, Fuzhou, China; 3 Fujian Key Laboratory of Tumor Microbiology, Fujian Medical University, Fuzhou, China

**Keywords:** Apoptosis, CDK5, Chemoresistance, Gastric cancer

## Abstract

**Background:**

Chemoresistance is a major clinical challenge that leads to tumor metastasis and poor clinical outcome. The mechanisms underlying gastric cancer resistance to chemotherapy are still unclear.

**Methods:**

We conducted bioinformatics analyses of publicly available patient datasets to establish an apoptotic phenotype and determine the key pathways and clinical significance. In vitro cell models, in vivo mouse models, and numerous molecular assays, including western blotting, qRT-PCR, immunohistochemical staining, and coimmunoprecipitation assays were used to clarify the role of factors related to apoptosis in gastric cancer in this study. Differences between datasets were analyzed using the Student’s t-test and two-way ANOVA; survival rates were estimated based on Kaplan–Meier analysis; and univariate and multivariate Cox proportional hazards models were used to evaluate prognostic factors.

**Results:**

Bulk transcriptomic analysis of gastric cancer samples established an apoptotic phenotype. Proapoptotic tumors were enriched for DNA repair and immune inflammatory signaling and associated with improved prognosis and chemotherapeutic benefits. Functionally, cyclin-dependent kinase 5 (CDK5) promoted apoptosis of gastric cancer cells and sensitized cells and mice to oxaliplatin. Mechanistically, we demonstrate that CDK5 stabilizes DP1 through direct binding to DP1 and subsequent activation of E2F1 signaling. Clinicopathological analysis indicated that CDK5 depletion correlated with poor prognosis and chemoresistance in human gastric tumors.

**Conclusion:**

Our findings reveal that CDK5 promotes cell apoptosis by stabilizing DP1 and activating E2F1 signaling, suggesting its potential role in the prognosis and therapeutic decisions for patients with gastric cancer.

**Supplementary Information:**

The online version contains supplementary material available at 10.1186/s12935-023-03112-4.

## Background

Gastric cancer is the fifth most common malignancy and fourth leading cause of cancer-related deaths globally [1]. Due to atypical symptoms in the early stage and inadequate screening, most cases of gastric cancer are diagnosed as advanced disease and are associated with a poor 5-year overall survival (OS) rate [2]. As a classic therapeutic strategy, the chemotherapy–surgery–chemotherapy model strikingly improves clinical outcomes in patients with gastric cancer [3, 4]. Unfortunately, development of acquired drug resistance in cancer cells is one of the major obstacles to the effective treatment of gastric cancer. The complicated mechanisms underlying drug resistance include the presence of cancer stem cells, enhancement of DNA damage repair, and inactivation of the apoptosis signaling pathway [5, 6].

Apoptosis is a highly ordered cell death process that is triggered and modulated through death receptor-mediated extrinsic pathways and mitochondrial-mediated intrinsic pathways [7, 8]. These apoptotic pathways are precisely regulated by a series of pro- and antiapoptotic molecules, whereas disruption of the balance of apoptosis has been linked to tumor initiation, progression, metastasis, and therapeutic resistance [9, 10]. A comprehensive understanding of the molecular mechanisms underlying apoptosis is essential for developing novel effective therapeutic strategies.

As an important member of the protein kinase family, cyclin-dependent kinase 5 (CDK5) participates in cell-type–specific and context-dependent opposing functions. CDK5 has been established as an oncogene in glioblastoma and breast, prostate, and cervical cancers [11–14]. Conversely, increasing evidence suggests a proapoptotic role for CDK5 in some human cell types [15–17] and a role in suppressing tumorigenesis in several human cancers [18–25]. The exact function of CDK5 in gastric cancer remains largely unknown. In this study, we analyzed differentially expressed genes (DEGs) across established apoptotic phenotypes and identified CDK5 as a proapoptotic gene in gastric cancer. We demonstrate that CDK5 promotes apoptosis in gastric cancer cells by directly binding to DP1 and subsequently activating E2F1 signaling. Our findings suggest that CDK5 may serve as a biomarker for the prognosis and therapeutic decisions in patients with gastric cancer.

## Methods

### Gastric cancer tissues and tissue microarrays

Gastric cancer tissues were obtained from Fujian Medical University Union Hospital (FJMUUH; Fuzhou, Fujian, China). A total of 340 gastric tumor tissues from three tissue microarrays (TMAs) and 97 adjacent nontumor tissues from one TMA were used for immunohistochemical (IHC) staining (Additional file 1: Table S1). Complete clinical, pathological, and follow-up data were available for most of these patients. In addition, to examine the correlation between CDK5 expression and chemotherapy response, gastric tumor tissues were collected from 19 patients who received neoadjuvant chemotherapy (Additional file 2: Table S2), and the expression level of CDK5 was assessed using western blotting assays. Therapeutic responses were evaluated according to RECIST 1.1. The study was approved by the Ethics Committee of FJMUUH, and informed consent was obtained from all enrolled patients.

### Public dataset collection and collation

To investigate the apoptotic phenotype and clinical molecular characteristics of gastric cancer, normalized RNA-sequencing data (as transcripts per million [TPM]) coupled with the associated clinical information in the gastric cancer samples were downloaded from the UCSC Xena browser (Genomic Data Commons [GDC] hub://gdc.xenahubs.net). The microarray datasets GSE27342, GSE65801, GSE79973, GSE13195, GSE118916, GSE130823, GSE66229 (Asian Cancer Research Group [ACRG] cohort), GSE26942, GSE84433, GSE15459, GSE122401, and GSE54129 were obtained from the Gene Expression Omnibus (GEO, https://www.ncbi.nlm.nih.gov/gds/), along with the relevant clinicopathological data and follow-up and chemotherapeutic information. Annotation files were used to match each probe from the microarray datasets to gene symbols. If a gene contained multiple probes, the mean value was used as the expression value.

### DEG analysis

To identify the genes that play a pivotal role in apoptosis during gastric cancer development, we performed DEG analysis of the apoptotic phenotypes in The Cancer Genome Atlas (TCGA), ACRG, and GSE26942 cohorts, using the “limma” R package and setting the significance levels of adjusted *p* value at < 0.01. The strategy for DEG screening comprised two steps: primary DEGs were identified by overlapping groups of DEGs from Cluster B vs. A, Cluster C vs. A, and Cluster C vs. B in the three gastric cancer cohorts; the final DEGs were identified by merging the three cohorts of pro/antiapoptotic primary DEGs. Venn diagrams were generated to present the number of overlapping genes. Through this strategy, we focused on particular genes with gradual up- or downregulated differential expression during apoptosis.

### Gene set enrichment analysis (GSEA)

We performed GSEA to explore the molecular mechanisms related to the apoptotic phenotype or those underlying CDK5 function. The TCGA and GEO samples were divided into high-, intermediate-, and low-expression groups based on the two cutoff values (mean ± standard deviation) of CDK5 gene expression. The samples with high vs. those with low CDK5 expression in the TCGA and GEO cohorts were included in the DEG analysis using the “limma” R package. GSEA was performed using the “clusterProfiler” R package [26] to calculate the normalization enrichment score (NES) and the false discovery rate (FDR) of each gene set. The reference gene sets, including hallmark and Gene Ontology (GO) biological process gene sets, were downloaded from the Molecular Signatures Database (MSigDB) of the Broad Institute (https://www.gsea-msigdb.org/). Gene sets with an FDR of less than 25% were considered significant. We generated heatmaps to display the NES for each gene set.

### Single-sample gene set enrichment analysis (ssGSEA)

To examine the enrichment score of a gene set for individual samples, we performed ssGSEA [27] for each sample in all gastric cancer cohorts using the R package GSVA. The epithelial–mesenchymal transition (EMT) and cytotoxic T-cell gene sets were obtained from previous publications [28, 29].

### Prediction of drug sensitivity

The half-maximal inhibitory concentrations (IC50) of oxaliplatin and docetaxel against each gastric cancer sample were predicted using the “oncoPredict” package in R based on the gene expression profiles in the GSE26942 dataset. The training datasets for drug sensitivity prediction were downloaded from the Genomics of Drug Sensitivity in Cancer resource (GDSC, https://www.cancerrxgene.org/).

### Unsupervised clustering

To establish the apoptotic phenotype in bulk samples, we collected 14 apoptotic signatures (Additional file 3: Table S3) from MSigDB. GSEA was performed on tumor samples from the TCGA and GEO cohorts compared with adjacent nontumor samples. We identified three key apoptotic signatures—Wu_TP53_apoptosis [30], Intrinsic_apoptosis, and Alcala_apoptosis [31]— based on their consistent NES across all datasets. We determined the optimal number of clusters as three by applying the “NbClust” algorithm (min.nc = 2, max.nc = 15, method=“kmeans”) using the “NbClust” R package. Lastly, we performed unsupervised clustering (K-means; centers = 3, nstart = 25) based on the ssGSEA scores of the three apoptotic signatures using “cluster” R packages.

### Cell lines, culture, and reagents

Human gastric cancer cell lines (AGS, MKN45, HGC-27, and MKN1) were purchased from CellCook Biotech Co. Ltd. (Guangzhou, Guangdong, China) and cultured in a humidified incubator under a 5% CO_2_ atmosphere at 37 °C. The MKN1 and MKN45 cells were maintained in RPMI 1640 medium (Gibco, Carlsbad, CA, USA). The AGS cells were cultured in F-12 K medium (Gibco). The HGC-27 cells were cultured in Dulbecco’s modified Eagle’s medium/Nutrient Mixture F-12 medium (DMEM/F-12; Gibco). All media were supplemented with 10% (v/v) fetal bovine serum (FBS; Gibco) and 1% (w/v) penicillin/streptomycin (Biosharp, Hefei, Anhui, China). All cultured cell lines were tested monthly using a MycoAlert™ Mycoplasma Detection Kit (LT07-418; Lonza Group, Basel, Switzerland). Cells were treated with oxaliplatin (S1224; Selleckchem; 0.25, 0.5 or 1.0 µM) for the indicated time points. Details of the reagents and kits used are listed in Additional file 4: Table S4.

### Protein isolation and western blotting

The proteins were extracted using RIPA lysis buffer (Beyotime, Beijing, China) containing phenylmethylsulfonyl fluoride (PMSF) (GenStar, Beijing, China). Protein concentration was determined using a BCA protein assay kit (Thermo Scientific, Waltham, MA, USA). The following primary antibodies were used: mouse anti-CDK5 (1:1000; sc-6247) and mouse anti-DP1 (1:1000; sc-53,642), purchased from Santa Cruz Biotechnology (Dallas, TX, USA). Rabbit anti-E2F-1 (1:1000; 3742 S), rabbit anti-apoptotic protease activating factor 1 (APAF1; 1:1000; 8969 S), and rabbit anti-p73 (1:1000; 14,620 S) antibodies were purchased from Cell Signaling Technology (Danvers, MA, USA). Antibodies against apoptosis-associated proteins such as caspase 3, cleaved caspase 3, poly-(ADP-ribose) polymerase (PARP), and cleaved PARP were obtained from Affinity Biosciences (1:1000; OH, USA). An ImageQuant LAS 4000 mini-instrument (GE Healthcare, Piscataway, NJ, USA) was used for signal detection. Detailed antibody information is provided in Additional file 4: Table S4.

### RNA extraction and quantitative real-time PCR (qRT-PCR)

Total RNA was isolated from the cell lines using TRIzol reagent (Invitrogen, Carlsbad, CA, USA). cDNA was prepared from 800 ng of total RNA using the PrimeScript RT Master Mix (Takara, Dalian, China) in accordance with the manufacturer’s instructions. mRNA expression was assessed using SYBR Green PCR Master mix (Takara) on a real-time PCR instrument (Bio-Rad, Hercules, CA, USA). The following primers were used in the present study: E2F1, forward: 5′-TGGTGATCAAAGCCCCTCCT-3′ and reverse: 5′-GGAAAACATCGATCGGGCCT-3′; DP1, forward: 5′-TCTAACGGCACAAGGTTCTCT-3′ and reverse: 5′-TCAGTCGTCCTCGTCATTCTC-3′; and GAPDH, forward: 5′- GGTGTGAACCATGAGAAGTATGA-3′ and reverse: 5′- GAGTCCTTCCACGATACCAAAG-3′.

### IHC staining

Gastric cancer and adjacent nontumor tissues were fixed with formalin, embedded in paraffin, and serially sectioned at a thickness of 4 μm. The sections were deparaffinized with dimethyl benzene and rehydrated in a graded series of ethanol solutions. Sodium citrate buffer (MXB Biotechnologies, Fuzhou, Fujian, China) was used to retrieve the antigens, and endogenous peroxidase activity was blocked with 3% hydrogen peroxide (MXB Biotechnologies). The heterogenetic antigen was blocked with 10% goat serum (Gibco) and incubated with an anti-CDK5 antibody (ab40773; Abcam, Cambridge, MA, USA) at 4 °C overnight. Subsequently, the sections were incubated with a secondary antibody (Zhongshan Biotechnology, Beijing, China) and developed in diaminobenzidine (DAB) solution (Zhongshan Biotechnology), followed by hematoxylin staining (Beyotime). A Motic EasyScan scanner (Hong Kong, China) was used to scan and analyze all the slides. The scoring criteria for IHC staining have been previously described [32]. Detailed antibody information is included in Additional file 4: Table S4.

### Flow cytometry for quantification of apoptosis

For the flow cytometry assay, 5 × 10^5^ cells were plated in 6-well plates and transfected with a CDK5 overexpression plasmid (oeCDK5) or control vector, purchased from GeneChem Corporation (Shanghai, China). After 48 h, the cells were harvested and washed twice with cold phosphate-buffered saline (PBS). The cells were stained with PE Annexin V Apoptosis Detection Kit I (559,763; BD Biosciences, Bedford, MA, USA) and examined on an LSRFortessa X-20 flow cytometer (BD Biosciences).

### Establishment of CDK5-overexpressing stable cell lines

To establish stable CDK5 overexpression clones, MKN45 cells were infected with lentivirus (control vehicle or oeCDK5 plasmid) in 8 µg/mL polybrene (TR-1003, Sigma–Aldrich). After 72 h of infection, the cells were treated with puromycin for two weeks to select stable CDK5-overexpressing cells, which were then examined using western blotting.

### Immunofluorescence (IF) assay

Gastric cancer cells were grown on 35-mm glass bottom dishes (D35-20-1-N; Cellvis) and incubated with 4% paraformaldehyde in PBS for 20 min, followed by permeabilization with 0.1% Triton X-100 in PBS for 10 min. After washing twice with PBS, the cells were blocked with 5% bovine serum albumin (BSA) for 30 min at 37 °C and incubated with rabbit anti-CDK5 (1:100; ab40773) and mouse DP1 (1:50; Santa Cruz Biotechnology) antibodies overnight at 4 °C. On the second day, the cells were washed with PBS and then incubated with goat anti-mouse IgG H&L (1:200; ab175473; Abcam) and goat anti-rabbit IgG H&L (1:200; ab150079; Abcam) for 30 min at 37 °C, followed by staining with 4′,6-diamidino-2-phenylindole (DAPI). Fluorescent images were acquired using a confocal laser scanning microscope (LSM 780, Carl Zeiss).

### Cell viability assay

The viability of the gastric cancer cells was evaluated using a Cell Counting Kit-8 (CCK-8; Dojindo, Kumamoto, Japan) assay. Briefly, 1 × 10^5^ AGS and MKN45 cells in culture medium were plated in each well. The cells were divided into control and overexpression groups. Each well was incubated with gradual concentrations of oxaliplatin at 37 °C. After 72-h treatment, the cells were incubated for 2 h with 10% CCK-8 reagent. The OD at 490 nm was measured. IC50 was calculated using GraphPad Prism 9 (GraphPad Software, La Jolla, CA, USA). Detailed reagent and kit information is included in Additional file 4: Table S4.

### Caspase 3 activity assay

The caspase 3 activity was detected using GreenNuc™ caspase-3 activity detection kit (C1168M; Beyotime, China). Briefly, 1 × 10^4^ of AGS, MKN45, HGC-27 and MKN1 cells with CDK5 overexpression and/or E2F1 knockdown were seeded in chamber slide and, were allowed to grow overnight in a humidified incubator under a 5% CO_2_ atmosphere at 37 °C. After incubating for 48 h, the cells were incubated for 30 min with GreenNuc reagent. Fluorescent images were acquired using a confocal laser scanning microscope (LSM 780, Carl Zeiss).

### Coimmunoprecipitation (coIP) assay

The cells were washed with PBS and lysed using IP lysis buffer (Beyotime). The lysates were incubated with the indicated antibodies overnight at 4 °C, following by incubation with Protein A/G Plus-Agarose (SC-2003; Santa Cruz Biotechnology) at 4 °C for another 4 h. The immunocomplex was washed 4–6 times with IP lysis buffer, added to 2× sodium dodecyl sulfate (SDS) sample buffer and boiled for 10 min at 99 °C. The immunoprecipitated proteins were separated using SDS–polyacrylamide gel electrophoresis (PAGE), followed by incubation with specific antibodies and detection using western blotting.

### Protein stability assay

The inhibitor of protein synthesis cycloheximide (CHX) was used to assess the stability of the DP1 protein affected by the CDK5 status. The cells were treated with 20 µM CHX in the presence or absence of 10 µM proteasome inhibitor MG-132 for the indicated times. Protein expression was evaluated using western blotting. Details of the reagents and kits are listed in Additional file 4: Table S4.

### Ubiquitination assay

To evaluate in vitro the role of CDK5 in DP1 degradation by ubiquitination, MKN45 cells were treated with MG-132 (10 mmol/L; MedChemExpress, Shanghai, China) for 6 h. The total cell lysates were immunoprecipitated with an anti-DP1 antibody overnight and subsequently rotated and incubated with protein A/G beads for 2–4 h at 4 °C. The beads were washed with IP lysis buffer 4–6 times, mixed with 2× SDS sample buffer and boiled for 10 min at 99 °C. The immunoprecipitated proteins were analyzed using SDS–PAGE, followed by incubation with the indicated antibodies and detection using western blotting.

### In vivo xenograft mouse model

Female BALB/c nude mice (4–5 weeks of age) were purchased from Beijing Vital River Laboratory Animal Technology Co., Ltd. (China). All experiments involving animals were approved by the Ethics Committee of Fujian Medical University/Laboratory Animal Center (Fuzhou, Fujian, China). All animal experiments were performed in accordance with the guidelines of the Animal Protection Committee of Fujian Medical University (Fuzhou, Fujian, China). Vehicle control and CDK5 overexpression MKN45 cells (1 × 10^6^) were injected subcutaneously into the left flank of each mouse. One week after inoculation, the mice were randomly assigned to four groups (n = 5 tumors per group): (a) vehicle (CTRL); (b) oxaliplatin treatment (CTRL + OXA); (c) CDK5 overexpression (oeCDK5); and (d) a combination of oxaliplatin treatment and CDK5 overexpression (oeCDK5 + OXA). The mice were administered solution or oxaliplatin (2 mg/kg/3 d, intraperitoneally). The tumor size was monitored twice a week using a caliper. The tumor volume was calculated according to the following equation: V = (L × W^2^)/2 (V, tumor volume; L, length; and W, width).

### Statistical analysis

All statistical analyses were performed using the R software (R 3.6.1; https://www.r-project.org/) or GraphPad Prism 9. The differences between datasets were analyzed using Student’s t-test and two-way ANOVA. The data are presented as the means ± SEM. Associations between two categorical variables were examined using the chi-squared test. The Kaplan–Meier method was used to calculate survival rates, which were analyzed using the log-rank test. Univariate and multivariate Cox proportional hazards models were used to evaluate prognostic factors. ****p < 0.0001; ***p < 0.001; **p < 0.01; and *p < 0.05; ns, not significant.

## Results

### Apoptotic phenotype correlates with TCGA subtype and predicts survival in gastric cancer

**Fig. 1:**
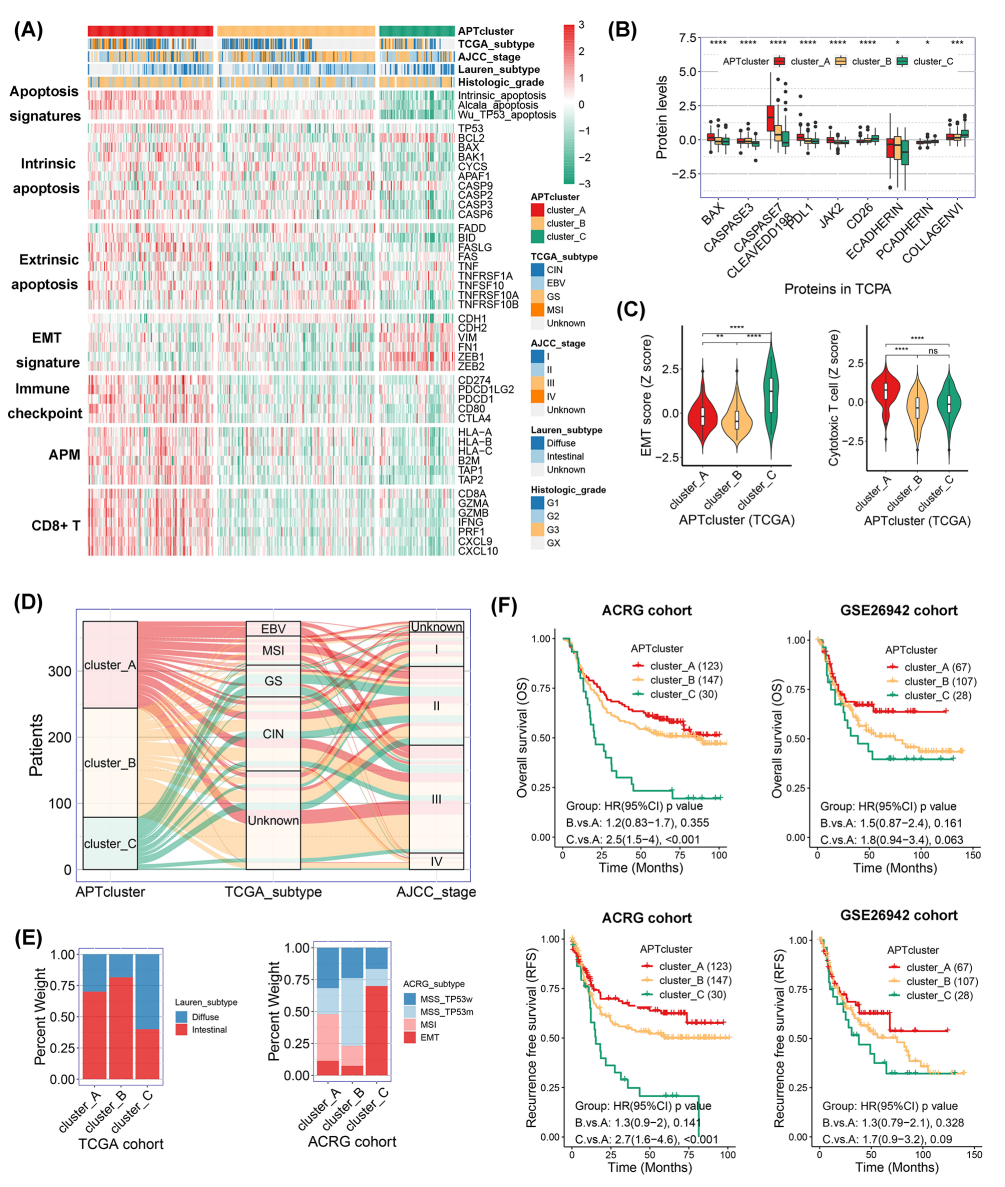
Apoptotic phenotype correlates with TCGA subtype and prognosis in gastric cancer. (**A**) Heatmap showing the expression of marker genes in several functional gene sets in The Cancer Genome Atlas (TCGA) gastric cancer cohort. (**B**) Relative protein levels in The Cancer Proteome Atlas (TCPA) cohort. *p < 0.05, ***p < 0.001, and ****p < 0.0001. (**C**) The violin plot displays the scores of epithelial–mesenchymal transition (EMT) and cytotoxic T-cell signatures calculated by single sample gene set enrichment analysis (ssGSEA). The values are normalized to the z score. Ns indicates no significance, **p < 0.01, and ****p < 0.0001. (**D** and **E**) Correlation of apoptotic phenotype (APTcluster) with clinicopathological characteristics in the TCGA or Asian Cancer Research Group (ACRG) cohorts displayed in a Sankey diagram (**D**) and percentage histogram (**E**). (**F**) Kaplan–Meier survival analysis for patients with different apoptotic phenotypes in the ACRG and GSE26942 cohorts

To investigate the intrinsic characteristics and function of apoptosis in the initiation and progression of gastric cancer, we performed GSEA using several public gastric cancer cohorts—TCGA, and seven GEO datasets. Three apoptosis signatures, Wu_TP53_apoptosis, Intrinsic_apoptosis, and Alcala_apoptosis, were consistently enriched in tumor tissues across all datasets compared to adjuvant nontumor tissues (Additional file 5: Figure S1A) and their presence was associated with a favorable survival prognosis for gastric cancer in the ACRG cohort (Additional file 5: Figure S1B and S1C). The apoptotic phenotype APTcluster was established based on the scores of the three apoptosis-related signatures in the three gastric cancer cohorts—TCGA, ACRG, and the GSE26942 dataset. Based on the analyses of data from all cohorts (Fig. 1A C, Additional file 6: Figure S2A and S2B), Cluster A was defined as a proapoptotic phenotype characterized by activation of both intrinsic and extrinsic apoptosis signatures and enrichment of the immune inflammatory signatures; this was evident in the elevated expression of immune checkpoints and antigen presentation machinery (APM) signature and high infiltration of CD8^+^ T effector cells. Cluster C was identified as a phenotype with obvious antiapoptotic signatures and significant enrichment for the EMT signature and immunosuppressive phenotype. Cluster B was an intermediate phenotype, with mixed pro- and antiapoptotic characteristics.

We next explored differences in the clinical features and prognostic value among the apoptotic phenotypes. The majority of Epstein–Barr virus (EBV)-associated and microsatellite instability (MSI) tumors were identified as proapoptotic, whereas most of the chromosomal instability (CIN) and genome stable (GS) subtypes were classified as intermediate and antiapoptotic phenotypes, respectively (Fig. 1D). Additionally, most diffuse-subtype gastric cancers and those with an EMT phenotype were enriched in the antiapoptotic phenotype (Fig. 1E). Lastly, poorer OS and recurrence-free survival (RFS) rates were observed for antiapoptotic tumors than for both intermediate and proapoptotic tumors in the TCGA and GEO cohorts (Fig. 1F, Additional file 6: Figure S2C). Taken together, our data indicate that the apoptotic phenotype established by clustering three apoptosis signatures correlated with the EMT phenotype and TCGA subtype and had prognostic value in gastric cancer.

### Proapoptotic tumors are enriched in DNA repair and immune inflammatory pathways and predict chemotherapeutic benefit

**Fig. 2:**
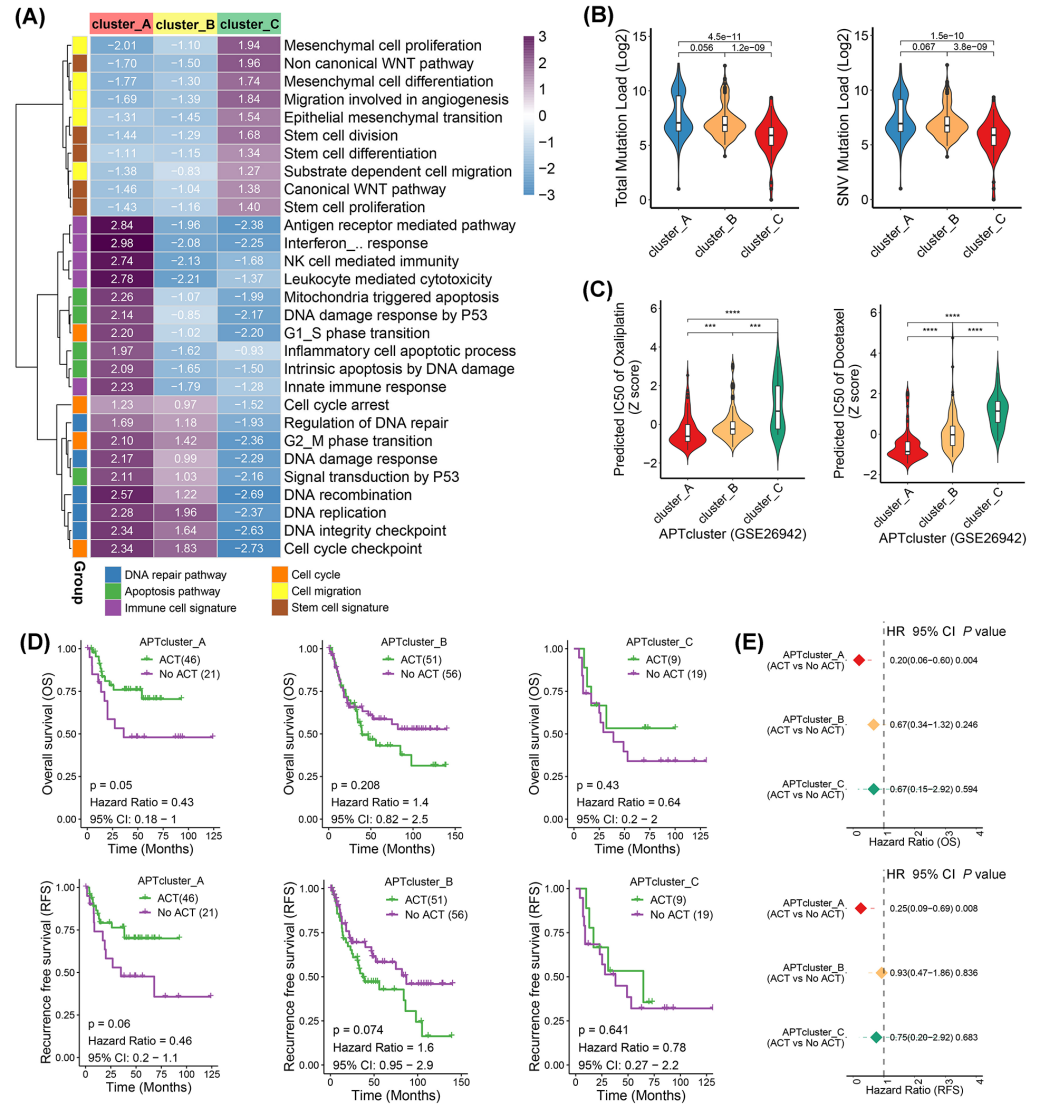
Proapoptotic tumors are enriched in DNA-repair and immune-inflammatory signaling pathways and predict chemotherapeutic benefits. (**A**) Gene set enrichment analysis (GSEA) was performed in the TCGA cohort using a panel of functional gene sets related to Gene Ontology (GO) biological processes and hallmark items from the Molecular Signatures Database (MSigDB) of the Broad Institute. The heatmap shows the normalized enrichment score (NES) for each gene set. (**B**) The violin plot displays total mutation load and single nucleotide variant (SNV) mutation load among apoptotic phenotypes in the TCGA cohort. The values were log2 transformed. (**C**) The violin plot presents the IC50 of oxaliplatin (OXA) and docetaxel assessed for each sample in the GSE26942 cohort using the OncoPredict algorithm. (**D**) Kaplan–Meier survival analysis for patients with and without adjuvant chemotherapy (ACT) treatment among different apoptotic phenotypes in the GSE26942 cohort. (**E**) Adjusted hazard ratio for patients who received ACT among different apoptotic phenotypes using Cox regression model compared with patients who did not receive ACT.

We next dissected the biological functions of apoptotic phenotypes using integrated pathway analysis. Three hundred and seventy-five tumor samples in the TCGA cohort were clustered into three apoptotic phenotypes based on the above algorithm: proapoptotic (cluster_A: 131 samples), intermediate (cluster_B: 165 samples), and antiapoptotic (cluster_C: 79 samples). We performed GSEA using a panel of functional signatures from MSigDB on samples from each phenotype versus those from the other phenotypes. We generated a heatmap based on the NES for each signature (Fig. 2A). Interestingly, the proapoptotic phenotype exhibited activated immune inflammatory signaling, including type I interferon response, antigen receptor-mediated pathway, and leukocyte-mediated cytotoxicity. Several notable pathways, including cell cycle signatures, DNA repair signatures, and the p53 pathway, were commonly enriched in the proapoptotic and intermediate phenotypes. Conversely, obvious activation of the cell migration pathway (including EMT and angiogenesis), canonical and noncanonical WNT pathways, and stem cell signatures (especially stem cell division and differentiation) were observed in the antiapoptotic phenotype (Fig. 2A, Additional file 7: Figure S3A).

We next investigated the molecular characteristics of the apoptotic phenotypes using genomic data associated with stomach cancer in the UCSC TCGA hub (https://tcga.xenahubs.net). In total, genomic data from 372 cases (130, 165, and 77 in cluster_A, cluster_B, and cluster_C, respectively) were obtained for this study. Consistent with the results of the Sankey plot (Fig. 1D), our findings revealed that the somatic mutation load, including total mutations, single nucleotide variants (SNV), and frame shifts and insertion/deletion mutations, gradually decreased from Cluster A to C (Fig. 2B, Additional file 7: Figure S3B); this result suggests that the level of chromosomal or genomic stability contributes to the apoptotic phenotype in gastric cancer. Relative mutation and copy number variation frequency associated with apoptosis signaling, receptor tyrosine kinase signaling, immune checkpoints, and EMT signaling were lower in the antiapoptotic than in the proapoptotic phenotype (Additional file 7: Figure S3C); this finding may provide a rational interpretation for the activation of apoptosis and therapeutic response in proapoptotic tumors.

Given that previous studies reported chemoresistance in gastric cancers with immunosuppressive or mesenchymal phenotypes [33, 34], we next sought to determine whether each apoptotic phenotype correlated with differences in the clinical benefit from adjuvant chemotherapy (ACT). We first predicted the IC50 of cisplatin for each sample in the GSE26942 cohort using the oncoPredict algorithm [35]. Our results indicated a higher IC50 for oxaliplatin and docetaxel in antiapoptotic than in proapoptotic tumors (Fig. 2C), suggesting that patients with the latter phenotype may be more sensitive to chemotherapy. To confirm the computational analysis, we examined the chemotherapy benefit in the GSE26942 cohort, in which more than half of the patients had received ACT. We found a significant increase in OS and RFS rates in patients with a proapoptotic phenotype (*p* = 0.05 for OS and *p* = 0.06 for RFS; Fig. 2D), whereas no benefit was observed in patients with intermediate or antiapoptotic phenotypes (all *p* > 0.05; Fig. 2D). This was further confirmed after adjusting for other clinicopathological features in the Cox regression model (Fig. 2E). Collectively, these results suggest that proapoptotic tumors harbor activated DNA repair and immune inflammatory signaling pathways and may be more sensitive to chemotherapy.

### *CDK5* is a potential proapoptotic gene in gastric cancer

**Fig. 3:**
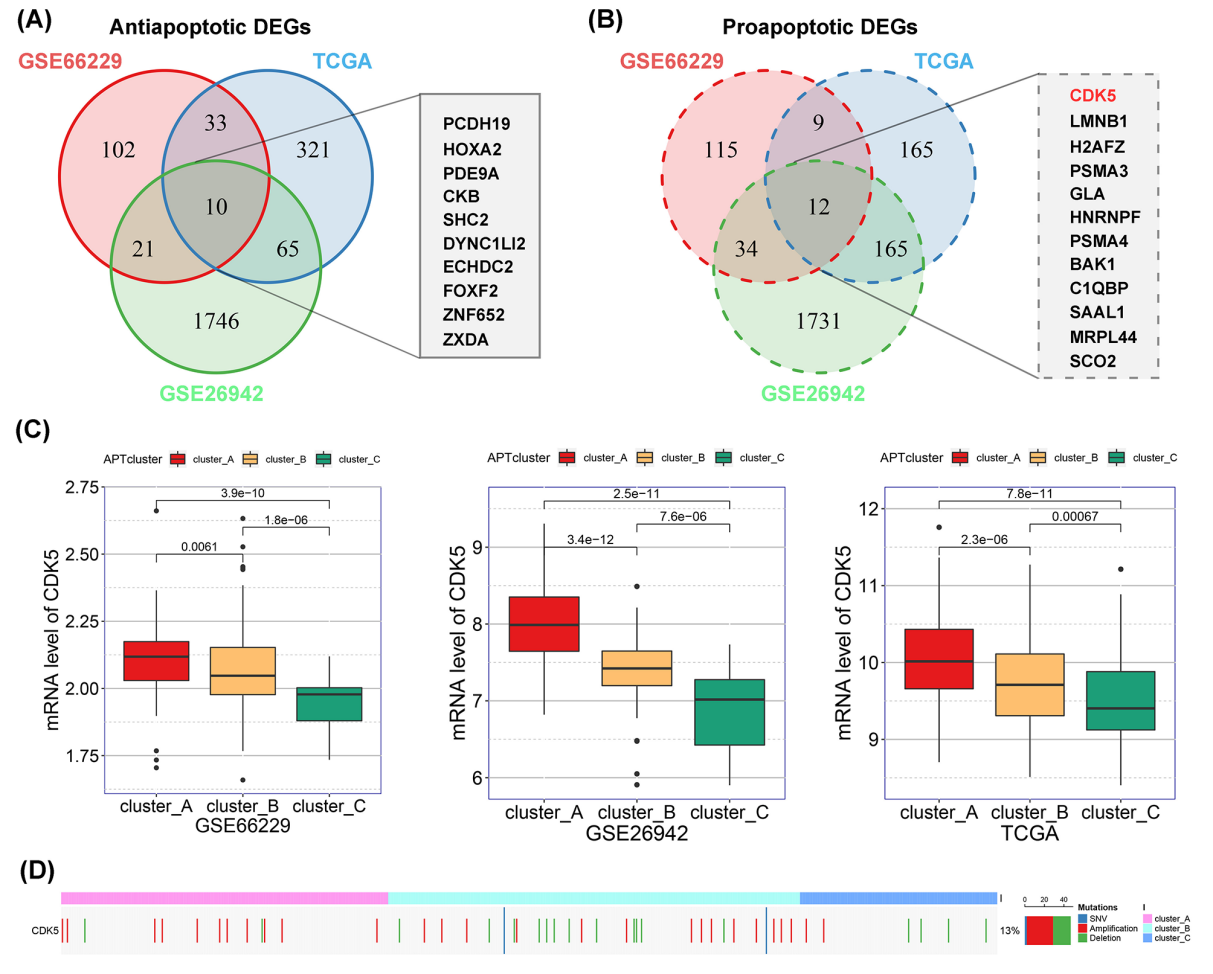
*CDK5* is a potential proapoptotic gene in gastric cancer. (**A** and **B**) Venn diagram shows differentially expressed genes (DEGs) among the apoptotic phenotypes from the TCGA, ACRG, and GSE26942 cohorts. Ten and 12 robust genes with differential expression were further identified by merging the three cohorts of anti- (**A**) or proapoptotic DEGs (**B**), respectively. (**C**) The histogram indicates that cyclin-dependent kinase 5 (*CDK5*) expression gradually increased in the proapoptotic (Cluster A), intermediate (Cluster B), and antiapoptotic phenotypes (Cluster C). (**D**) Heatmap showing copy number variations of *CDK5* in each apoptotic phenotype

To identify key genes that regulate apoptosis during the development of gastric cancer, we analyzed DEGs across the apoptotic phenotypes in the TCGA, ACRG, and GSE26942 cohorts. By overlapping the three groups of DEGs from Cluster B vs. A, Cluster C vs. A, and Cluster C vs. B, we obtained 166, 1842, and 429 antiapoptotic DEGs (Additional file 8: Figure S4A) and 170, 1942, and 351 proapoptotic DEGs (Additional file 8: Figure S4B) from the ACRG, GSE26942, and TCGA cohorts, respectively. Lastly, we combined the three cohorts of pro- and antiapoptotic DEGs and identified 10 and 12 genes with robust upregulated expression in the antiapoptotic and proapoptotic tumors, respectively (Fig. 3A and B). Intriguingly, of these proapoptotic genes, *CDK5* was shown to be deficient in gastric cancer and to suppress in vitro cancer cell proliferation and xenograft tumorigenesis in our previous study [18]. Here, we observed that, compared to antiapoptotic tumors, elevated expression of *CDK5* in proapoptotic tumors (Fig. 3C) may result from copy number variations but not from somatic mutations or promoter methylation (Fig. 3D, Additional file 8: Figure S4C and S4D). Thus, our results suggest that *CDK5* is a proapoptotic gene in gastric cancer.

### CDK5 promotes apoptosis and chemosensitivity in gastric cancer

**Fig. 4:**
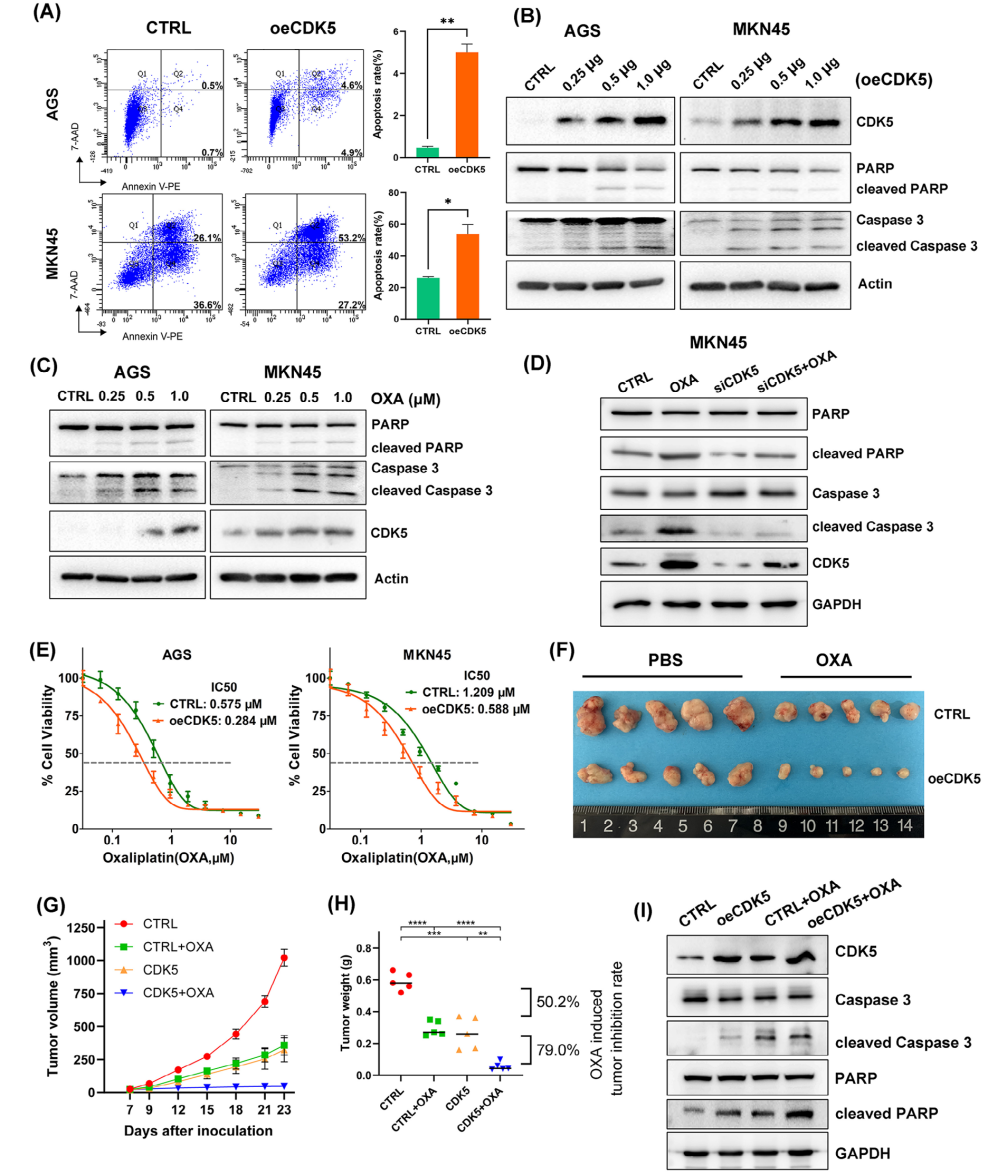
CDK5 promotes apoptosis and oxaliplatin-mediated chemosensitivity in gastric cancer. (**A**) Annexin V/PI staining for apoptosis assessed the apoptosis rates in AGS and MKN45 cells after reconstitution of CDK5. The quantification of apoptosis rates is shown as the mean ± SD of 3 independent experiments (right); *p < 0.05 and **p < 0.01. (**B**–**D**) Western blots of CDK5, total/cleaved poly-(ADP-ribose) polymerase (PARP), and total/cleaved caspase 3 in AGS and MKN45 cells. Panel B shows that the cells were transfected with the indicated amounts (0.25, 0.5 or 1.0 µg) of pLV-CDK5 expression plasmid or empty vector (CTRL). Panel C shows that the cells were treated with the indicated amounts (0.25, 0.5 or 1.0 µM) of oxaliplatin (OXA) or DMSO (CTRL). Panel D shows that the cells were treated with 0.5 µM OXA or siRNA targeting CDK5. (**E**) Cell viability assay results show the IC50 curves for oxaliplatin in the AGS and MKN45 cell lines with CDK5 overexpression. (**F**) Gross morphology of tumors in nude mice using MKN45 cells with CDK5 overexpression or combined with OXA treatment. The mice were dosed with OXA (2 mg/kg, intraperitoneally) (n = 5 tumors per group). (**G**) Tumor growth curve over time for each group. Data are shown as the mean ± SEM. (**H**) Relative tumor volume at the end of treatment. Lines in the graph indicate the median of tumor volume. **p < 0.01, ***p < 0.001, and ****p < 0.0001. (**I**) Western blots of CDK5, total/cleaved PARP, and total/cleaved caspase 3 from xenograft tumors

The above results indicate that CDK5 may regulate cell apoptosis in gastric cancer. To investigate whether CDK5 exerts its tumor-suppressive function in gastric cancer by activating apoptosis in tumor cells, we performed Annexin V/PI staining in the AGS and MKN45 cell lines to detect apoptosis. Reconstitution of CDK5 into tumor cells significantly increased apoptosis rates in both cell lines (all *p* < 0.05, Fig. 4A). In line with these results, CDK5 overexpression dramatically increased caspase 3 activity, as shown in the green staining in the four gastric cancer cell lines (Additional file 9: Figure S5A). Western blotting showed that transient overexpression of CDK5 obviously increased the expression of cleaved PARP and caspase 3 in gastric cancer cell lines (Fig. 4B and Additional file 9: Figure S5B), suggesting that intrinsic apoptosis is induced by CDK5. Interestingly, oxaliplatin treatment increased the levels of cleaved PARP and caspase 3 and promoted the expression of CDK5 in gastric cancer cell lines (Fig. 4C and Additional file 9: Figure S5C). In contrast, the accumulation of cleaved PARP and caspase 3 induced by oxaliplatin treatment was partially abrogated by CDK5 knockdown in MKN45, HGC-27, and MKN1 cells (Fig. 4D and Additional file 9: Figure S5D), implying that CDK5 plays a role in oxaliplatin -induced apoptosis.

Given the results obtained from the bioinformatics analysis that show high expression of CDK5 and its potential role in the regulation of proapoptotic tumors that exhibited sensitivity to chemotherapy, we tested whether CDK5 promoted chemosensitivity. We found that overexpression of CDK5 significantly sensitized gastric cancer cells to oxaliplatin treatment, as indicated by reductions in the IC50 values (Fig. 4E). An in vivo tumor xenograft model of CDK5 overexpression combined with oxaliplatin treatment was established to verify the in vitro findings of the cytotoxicity assay. The combination of increased CDK5 levels and oxaliplatin treatment repressed tumor growth by 79.0% compared to increased CDK5 levels alone, whereas oxaliplatin treatment alone moderately inhibited tumor growth in control cells (tumor inhibition rate = 50.2%) (Fig. 4F H). Moreover, western blotting confirmed that overexpression of CDK5 activated the intrinsic apoptotic pathway, as indicated by the increased levels of cleaved PARP and caspase 3, which possibly contributed to the inhibition of gastric tumor growth (Fig. 4I). Taken together, our in vitro and in vivo findings suggest that CDK5 significantly promoted apoptosis and sensitized gastric cancer cells to oxaliplatin.

### CDK5 induces apoptosis through direct binding to DP1 and activation of E2F1 signaling

**Fig. 5:**
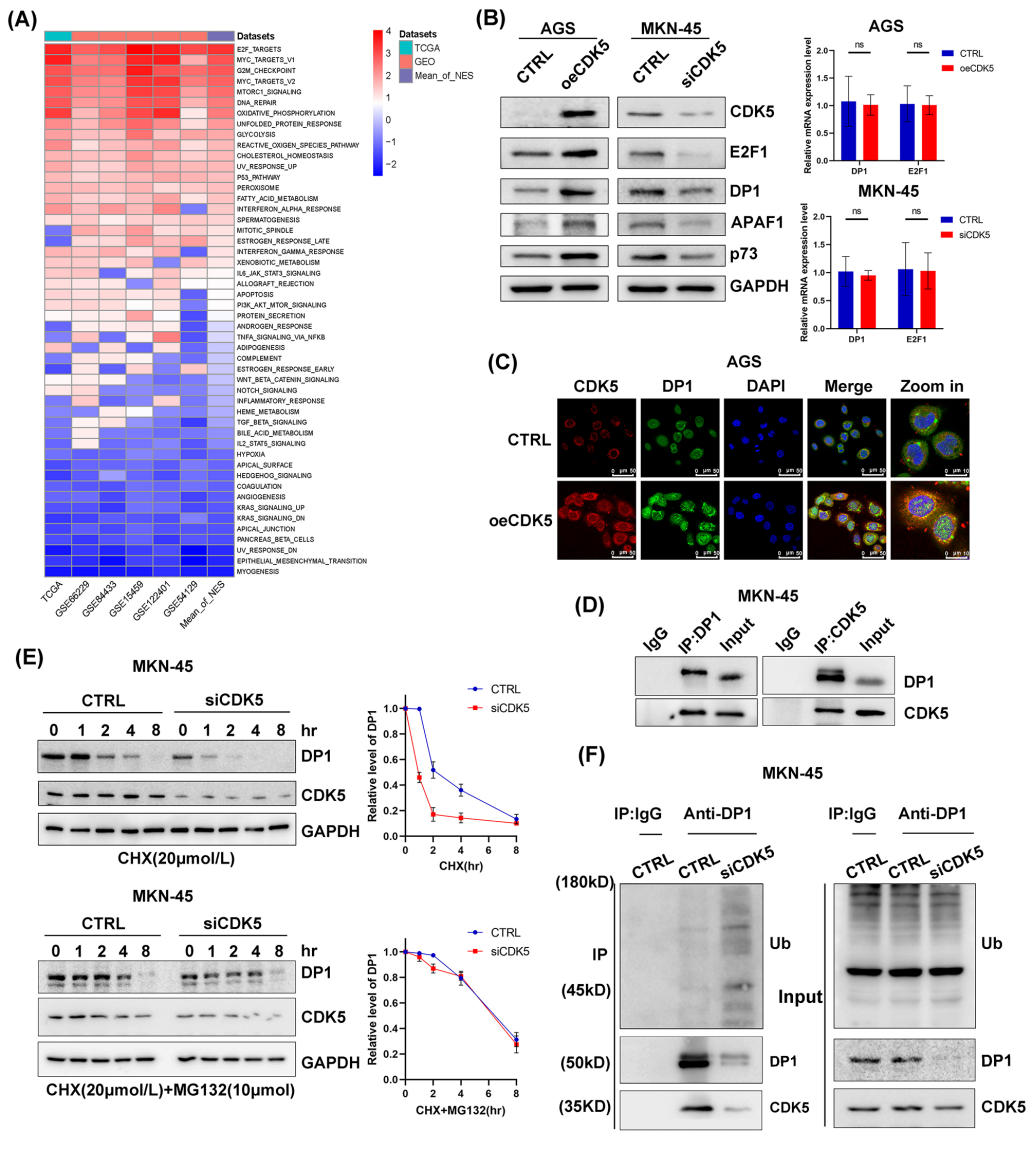
CDK5 induces apoptosis through direct binding to DP1 and activation of E2F1 signaling. (**A**) GSEA was performed in the TCGA and a panel of Gene Expression Omnibus (GEO) cohorts using 50 hallmark gene sets from MSigDB. Heatmap showing the NES for each gene set. (**B**) Western blots (left) of CDK5, E2F1, DP1, apoptotic protease activating factor 1 (APAF1), and p73 in AGS cells with CDK5 overexpression or MKN45 cells with CDK5 knockdown. qRT–PCR analysis (right) of *E2F1* and *DP1* in AGS cells with CDK5 overexpression or MKN45 cells with CDK5 knockdown. Ns indicates no significance. (**C**) Representative immunofluorescence images (scale bars, 50 μm) of CDK5 (red), DP1 (green) and 4′,6-diamidino-2-phenylindole (DAPI; blue) in CDK5-overexpressing AGS cells. (**D**) Immunoprecipitation (IP) assay using an antibody against CDK5 or DP1 in MKN45 cells. IgG was used as a negative control. Western blotting was performed for CDK5 and DP1. (**E**) Effects of CDK5 depletion on DP1 stability in MKN45 cells. The cells were incubated with cycloheximide (CHX) for the indicated times. (**F**) Effect of CDK5 on DP1 ubiquitination in MKN45 cells

To elucidate the downstream molecular mechanism of apoptosis regulated by CDK5 in gastric cancer, we performed GSEA in TCGA and several GEO cohorts by comparing *CDK5*-high-expression samples to *CDK5*-low-expression samples. Strikingly, a panel of pivotal functional gene sets, including E2F signaling, DNA repair signaling, and the p53 pathway, was consistently enriched across all cohorts, as shown in the heatmap (Fig. 5A). As the top signaling pathway enriched in the *CDK5*-high-expression samples, E2F signaling may be the downstream apoptotic pathway regulated by CDK5. To test this hypothesis, CDK5 was transiently overexpressed or silenced in gastric cancer cells. CDK5 overexpression upregulated the levels of E2F1 and DP1 and increased those of their downstream targets (APAF1 and p73) in AGS, HGC-27, and MKN1 cells. Conversely, CDK5 knockdown using siRNA markedly decreased E2F1 signaling in MKN45, HGC-27, and MKN1 cells compared with that in the respective control groups (Fig. 5B and Additional file 10: Figure S6A). However, there were no significant changes in the mRNA levels of *E2F1* and *DP1* (Fig. 5B and Additional file 10: Figure S6A). To test whether CDK5 directly induces apoptosis and chemosensitivity through the activation of E2F1 signaling, we examined the apoptosis and viability rates in AGS and HGC-27 cells. Western blotting showed that the increased expression of cleaved PARP and cleaved caspase 3 induced by CDK5 was abrogated by E2F1 knockdown (Additional file 10: Figure S6B). Similar results were observed in the caspase 3 activity assay (Additional file 10: Figure S6C). We further revealed that the increased sensitivity of tumor cells to oxaliplatin induced by CDK5 was restored by E2F1 knockdown (Additional file 10: Figure S6D), suggesting that CDK5-induced chemosensitivity is dependent on E2F1.

Given the posttranscriptional regulation of E2F1 and DP1 by CDK5, we next explored the potential mechanism of CDK5–DP1 signaling. IF staining showed that CDK5 overexpression elevated DP1 expression and enhanced its colocalization in the cytoplasm in AGS cells (Fig. 5C). Using IP, we detected a novel protein–protein interaction between CDK5 and DP1 (Fig. 5D). We also examined whether CDK5 affects DP1 protein stability. Our findings indicate that CDK5 depletion shortened the half-life of DP1 (Fig. 5E). The acceleration of DP1 degradation was reversed in the presence of the proteasome inhibitor MG-132, suggesting that CDK5 impedes DP1 degradation via the ubiquitin proteasome system (Fig. 5E). As expected, CDK5 silencing increased DP1 ubiquitination (Fig. 5F). Collectively, these data suggest that CDK5 stabilizes DP1 through direct binding with DP1 and the subsequent activation of E2F1 signaling.

### CDK5 depletion predicts poor survival and chemoresistance in patients with gastric cancer

**Fig. 6:**
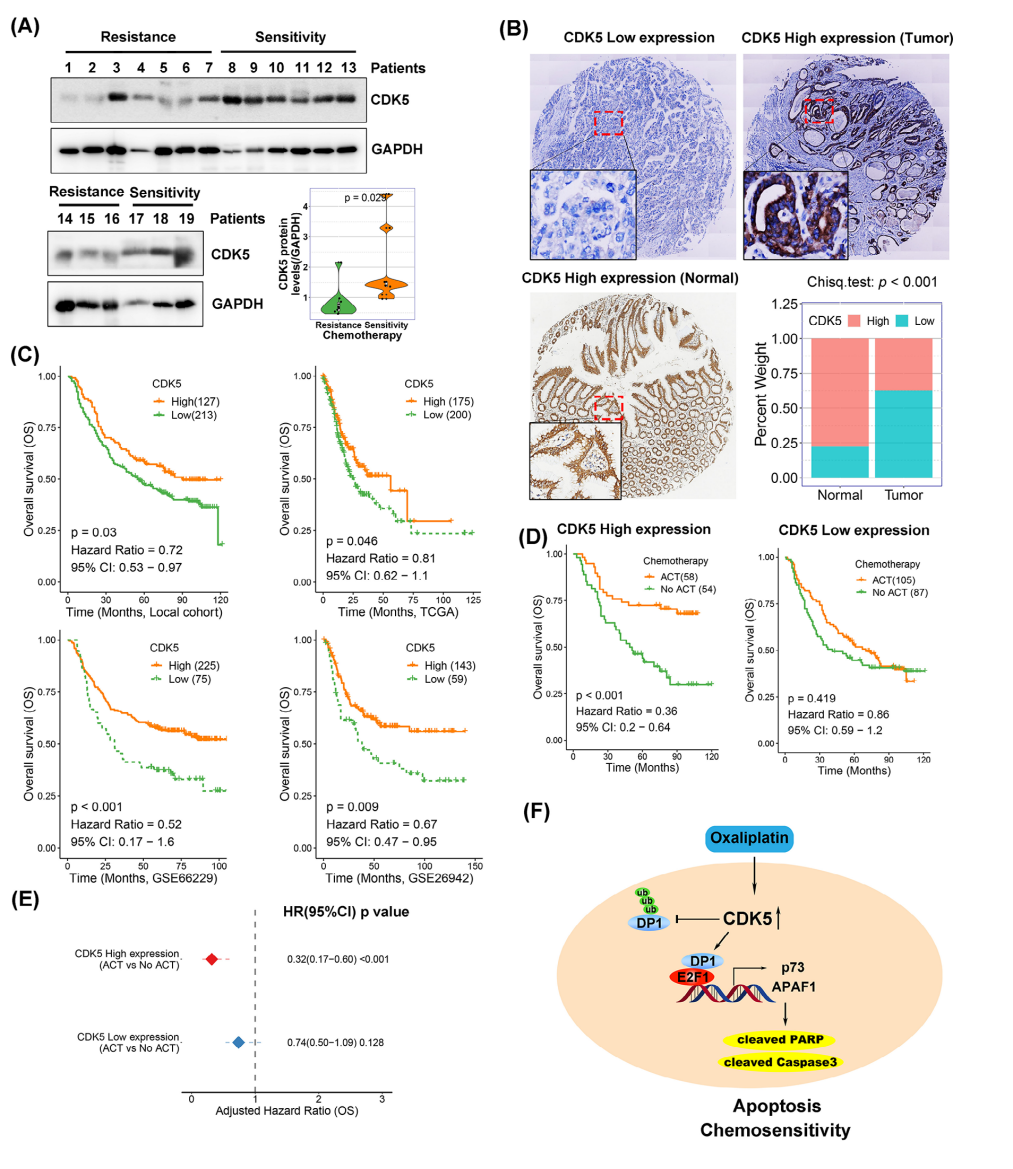
CDK5 depletion is associated with poor prognosis and chemoresistance in patients with gastric cancer. (**A**) Western blots of CDK5 in human gastric cancer with resistance or sensitivity to chemotherapy. Densitometric analysis of CDK5 expression (right bottom). (**B**) Representative immunohistochemical staining (scale bars, 100 μm) of CDK5 in human gastric tumors and adjacent nontumor tissues. Histogram showing percentages of CDK5 high and low staining in tumor and nontumor tissues (right bottom). (**C**) Kaplan–Meier survival analysis for CDK5 staining in the local cohort (upper left) or mRNA levels (bottom right) in the TCGA and GEO cohorts. (**D**) Kaplan–Meier survival analysis for patients with and without ACT treatment in CDK5 high- or low-expression cases in the local cohort. (**E**) Adjusted hazard ratio for patients who received ACT in the CDK5 high/low expression group using Cox regression compared with patients who did not receive ACT. (**F**) Schematic summary of the role of CDK5 in gastric cancer cell apoptosis. In brief, oxaliplatin-induced CDK5 stabilizes DP1 through direct binding with it and subsequent activation of E2F1 signaling, which promotes tumor cell apoptosis and sensitizes cancer cells to chemotherapy

Given the results from the cytotoxicity assay and tumor xenograft model that showed the capacity of CDK5 to promote chemosensitivity, we investigated the clinical value of CDK5 in gastric cancer. Using western blotting, we examined CDK5 protein levels in frozen gastric tumor tissues collected from 19 patients who received neoadjuvant chemotherapy (Additional file 2: Table S2). The results showed that CDK5 protein expression was significantly higher in patients who developed chemoresistance than in those with sensitivity (Fig. 6A, p < 0.05). In addition, we tested CDK5 expression using IHC staining in TMAs with 340 evaluable gastric cancer tissues and 97 evaluable adjacent nontumor tissues (Additional file 1: Table S1). Representative IHC staining for CDK5 low and high expression is shown in Fig. 6B. The histogram shows low CDK5 expression in 62.6% (213/340) of the tumor samples, which is remarkably higher than that in adjacent nontumor samples (22.7%, 22/97, *p* < 0.001; Fig. 6B). Kaplan–Meier survival analysis indicated a worse OS rate in the CDK5-low group (hazard ratio [HR] = 0.72, 95% confidence interval [CI]: 0.53–0.97, *p* = 0.03) than in the CDK5-high group (Fig. 6C). Similar results were obtained from analyses of the TCGA, ACRG, and GSE26942 cohorts (Fig. 6C; *p* < 0.05). More importantly, we assessed the survival benefit in our cohort of 304 patients with available information on platinum- and fluorouracil-based ACT (Additional file 1: Table S1). Our data demonstrated a significant survival benefit in the CDK5-high group who received ACT (HR = 0.36, 95% CI: 0.20–0.64, *p* < 0.001) compared to the CDK5-low group (HR = 0.86, 95% CI: 0.59–1.20, *p* = 0.419; Fig. 6D). This was further confirmed by adjusting for other clinicopathological features, including age, sex, tumor location, tumor size, and American Joint Committee on Cancer (AJCC) stage, in the Cox regression model (Fig. 6E). In summary, these results indicate that CDK5 depletion predicts poor survival and chemoresistance in patients with gastric cancer.

## Discussion

Gastric cancer is still a major clinical challenge due to resistance to therapies and poor overall prognosis [36, 37]. Dissecting the intrinsic characteristics of tumor molecular phenotypes is a key step toward the development of early diagnosis and effective therapeutic strategies. In this study, we present a simple method to detect an apoptotic phenotype with potential prognostic and therapeutic predictive value. Through DEG analysis of multiple phenotypes, we identified *CDK5* as a novel proapoptotic gene in gastric cancer. Our findings suggest CDK5 as a potential prognostic factor and molecular biomarker for chemotherapeutic decisions in gastric cancer.

Apoptosis, as a type of programmed cell death, is a physiological and pathological process that is pivotal for multicellular organisms to eliminate harmful cells and maintain cellular homeostasis [10]. The extrinsic (death receptor-mediated) and intrinsic (mitochondrial-mediated) pathways are two major apoptosis pathways that are precisely regulated by a series of proapoptotic molecules (such as BAX, BAK, BIM, BID, and PUMA) and antiapoptotic molecules (such as BCL-2, BCL-XL, and MCL1) [38, 39]. The balance of pro- and antiapoptotic proteins is considered a cellular rheostat for controlling cell death in mammalian cells [39, 40], whereas evasion of cell death due to disruption of this balance is one of the essential changes in tumorigenesis [41]. However, to date, comprehensive and integrated transcriptomic analyses that evaluate the balance between pro- and antiapoptotic signaling and the underlying mechanisms remain quite rare. In this study, we performed a transcriptomic analysis and established a valuable apoptotic phenotype based on the scores of three apoptosis signatures in gastric cancer. In line with previous evidence showing that various molecular signaling pathways are triggered during apoptosis [39, 42], our findings confirmed that the immune inflammatory phenotype was significantly enriched in proapoptotic tumors, whereas several critical oncogenic signaling pathways, including cell migration, WNT/β-catenin, and stem cell signatures, were activated in the antiapoptotic phenotype. Importantly, the apoptotic phenotype may serve as a novel predictive biomarker of clinical outcome and response to chemotherapy. Furthermore, the apoptosis signatures [30, 31] used in the current study were generated from different cancer cells, suggesting their potential universal application in various cancer types.

As an atypical member of the cyclin-dependent kinase family, the role of CDK5 has been well documented in the development, function, and pathologies of the central nervous system [43, 44]. Recently, accumulating evidence revealed that CDK5 has oncogenic functions in a variety of cancers, including colon, lung, breast, brain, pancreatic, and melanoma cancers [45–48]. However, our and other studies provide evidence that CDK5 exerts anti-tumor effects in various human cancers [18, 19, 49–53]. In terms of the function of CDK5 in apoptosis, previous findings have shown that CDK5 plays an antiapoptotic role in various disease types through different signaling pathways, including Fak-Akt [54] and Rb/E2F signaling [55]. Conversely, there is also sufficient evidence of its proapoptotic roles in various cells [16, 56]. Our results indicate that CDK5 promotes apoptosis through direct binding to DP1 and activation of E2F1 transcription. These opposing functions of CDK5 as anti- or proapoptotic protein may depend on the cell type and exogenous stimuli.

As a major clinical challenge that leads to tumor metastasis and poor clinical outcome, chemoresistance is a complex phenomenon responsible for failure to respond to chemotherapeutics [57]. Cancer cell resistance to chemotherapy can occur through different mechanisms, including epigenetic and genetic differences, alterations in drug metabolism, and inhibition of cell death (apoptosis suppression) [57, 58]. Our bioinformatics analysis indicated that patients with an antiapoptotic phenotype had worse OS and RFS rates than patients with proapoptotic tumors. Furthermore, in line with the results from the xenograft model, which showed that the exogenous expression of CDK5 rendered tumors more sensitive to oxaliplatin, our clinical data showed that high levels of CDK5 confer more survival benefits from ACT to patients than low CDK5 levels. Our data provide a scientific rationale for CDK5 as a potential biomarker of chemotherapeutic benefit.

## Conclusion

We propose an effective approach to reclassify gastric cancer into apoptotic phenotypes using transcriptomic data and discovered a CDK5-driven proapoptotic phenotype. Our findings demonstrate that CDK5 promotes gastric tumor cell apoptosis by directly binding to and stabilizing DP1 and activating E2F1 signaling. CDK5 depletion contributes to poor prognosis and chemoresistance in patients with gastric cancer (Fig. 6F). These findings suggest that CDK5 may serve as a potential biomarker for the prognosis and therapeutic decisions for patients with gastric cancer.

### Electronic supplementary material

Below is the link to the electronic supplementary material.

**Additional file 1**: Table S1: Clinicopathological characteristics and chemotherapy information of 340 patients with gastric cancer in TMAs.

**Additional file 2**: Table S2: Clinicopathological characteristics of 19 patients with gastric cancer.

**Additional file 3**: Table S3: Apoptotic signatures obtained from the MSigDB.

**Additional file 4**: Table S4: Reagents and Kits.

**Additional file 5**: Figure S1. Identification of three apoptosis signatures for the establishment of an apoptotic phenotype. (A) Heatmap showing GSEA results from a panel of public gastric cancer datasets, including the TCGA and several GEO cohorts. All apoptosis signatures were obtained from MSigDB. (B) Cox regression model analysis showing the hazard ratio for overall survival (OS) for each apoptosis signature in the TCGA cohort. (C) Kaplan–Meier survival analysis for patients with high or low ssGSEA scores of each signature in the ACRG cohort.

**Additional file 6**: Figure S2. Apoptotic phenotype correlates with TCGA subtype and is associated with prognosis in gastric cancer. (A and B) Heatmap showing the expression of marker genes in several functional gene sets in the ACRG (A) and GSE26942 (B) cohorts. (C) Kaplan–Meier survival analysis for patients with different apoptotic phenotypes in the TCGA cohort.

**Additional file 7**: Figure S3. Proapoptotic tumors are enriched in DNA-repair and immune-inflammatory signaling pathways and predict chemotherapeutic benefits. (A) GSEA was performed in the TCGA cohort using a panel of functional gene sets related to GO biological processes and hallmark items from MSigDB. The values indicate NESs. (B) Frame shift mutation load and frame insertion mutation load were examined among the apoptotic phenotypes in the TCGA cohort. The values were log2 transformed. (C) Somatic mutation and copy number variations were examined for apoptosis signaling, receptor tyrosine kinase signaling, immune checkpoints, and EMT signaling.

**Additional file 8**: Figure S4. *CDK5* is a potential proapoptotic gene in gastric cancer. (A and B) DEG analysis of the apoptotic phenotypes was performed in the TCGA, ACRG, and GSE26942 cohorts. Panel A shows 166, 1842, and 429 overlapping DEGs in the antiapoptotic phenotype, and panel B shows 170, 1942, and 351 overlapping DEGs in the proapoptotic phenotype. (C) Copy number variations of CDK5 were examined for each phenotype. (D) Promoter methylation of CDK5 was examined in each phenotype.

**Additional file 9**: Figure S5. CDK5 promotes apoptosis in gastric cancer. (A) Staining (scale bars = 50 μm) for apoptosis assessed caspase 3 activities in the four gastric cancer cell lines after overexpression of CDK5. Green staining indicates caspase 3 activity. (B–D) Western blotting of CDK5, total/cleaved PARP, and total/cleaved caspase 3 in HGC-27 and MKN1 cells. Panel B shows that cells were transfected with the indicated amounts (0.25, 0.5 or 1.0 µg) of pLV-CDK5 expression plasmid (oeCDK5) or empty vector (CTRL). Panel C shows that cells were treated with the indicated amounts (0.25, 0.5 or 1.0 µM) of oxaliplatin (OXA) or DMSO (CTRL). Panel D shows that cells were treated with 0.5 µM OXA or siRNA targeting CDK5.

**Additional file 10**: Figure S6. CDK5 induces apoptosis through activation of E2F1 signaling. (A) Western blotting (left) of CDK5, E2F1, DP1, APAF1, and p73 and qRT–PCR analysis (right) of *E2F1* and *DP1* in HGC-27 and MKN1 cells with CDK5 overexpression or knockdown. (B) Western blots of CDK5, E2F1, total/cleaved PARP, and total/cleaved caspase 3 in HGC-27 and AGS cells transfected with oeCDK5 plasmid and/or siRNA targeting E2F1. (C) Staining (scale bars = 50 μm) for apoptosis examined caspase 3 activity in AGS cells transfected with oeCDK5 plasmid and/or siRNA targeting E2F1. Green staining indicates caspase 3 activity. (D) Cell viability assay evaluated the IC50 curves for oxaliplatin in AGS and HGC-27 cells with CDK5 overexpression and E2F1 silencing.

## Data Availability

The dataset analyzed in this study is available from the corresponding author upon reasonable request.

## Abbreviations

ACRG: Asian Cancer Research Group
ACT: adjuvant chemotherapy
AJCC: American Joint Committee on Cancer
APAF1: apoptotic protease activating factor 1
APM: antigen presentation machinery
BSA: bovine serum albumin
CDK5: cyclin-dependent kinase 5
CHX: cycloheximide
CI: confidence interval
CIN: chromosomal instability
DAB: diaminobenzidine
DAPI: 4′,6-diamidino-2-phenylindole
DEG: differentially expressed gene
EBV: Epstein–Barr virus
EMT: epithelial–mesenchymal transition
Experimental groups: CTRL: vehicle; CTRL + OXA: oxaliplatin treatment; oeCDK5: CDK5 overexpression; and oeCDK5 + OXA: combination of oxaliplatin treatment and CDK5 overexpression
FDR: false discovery rate
FJMUUH: Fujian Medical University Union Hospital
GDC: Genomic Data Commons
GDSC: Genomics of Drug Sensitivity in Cancer
GEO: Gene Expression Omnibus
GO: Gene Ontology
GS: genome stable
GSEA: Gene set enrichment analysis
HR: hazard ratio
IC50: half-maximal inhibitory concentration
IF: immunofluorescence
IHC: immunohistochemical
IP: immunoprecipitation
L: tumor length
MSI: microsatellite instability
MSigDB: Molecular Signatures Database
NES: normalization enrichment score
ns: not significant
OS: overall survival
PARP: poly-(ADP-ribose) polymerase
PBS: phosphate-buffered saline
PMSF: phenylmethylsulfonyl fluoride
qRT-PCR: Quantitative real-time PCR
RFS: recurrence-free survival
SDS–PAGE: sodium dodecyl sulfate–polyacrylamide gel electrophoresis
SNV: single nucleotide variant
ssGSEA: single sample gene set enrichment analysis
TCGA: The Cancer Genome Atlas
TMAs: tissue microarrays
TPM: transcripts per million
V: tumor volume
W: tumor width
