# Supplementary material for: CDK5 promotes apoptosis and attenuates chemoresistance in gastric cancer via E2F1 signaling
Source: Cancer Cell Int. 2023 Nov 21;23:286. doi: 10.1186/s12935-023-03112-4 (PMC10664659; doi:10.1186/s12935-023-03112-4)
Supplement: Supplementary file 5 — Additional file 5: Figure S1. Identification of three apoptosis signatures for the establishment of an apoptotic phenotype. (A) Heatmap showing GSEA results from a panel of public gastric cancer datasets, including the TCGA and several GEO cohorts. All apoptosis signatures were obtained from MSigDB. (B) Cox regression model analysis showing the hazard ratio for overall survival (OS) for each apoptosis signature in the TCGA cohort. (C) Kaplan–Meier survival analysis for patients with high or low ssGSEA scores of each signature in the ACRG cohort. [file 12935_2023_3112_MOESM5_ESM.docx]

**
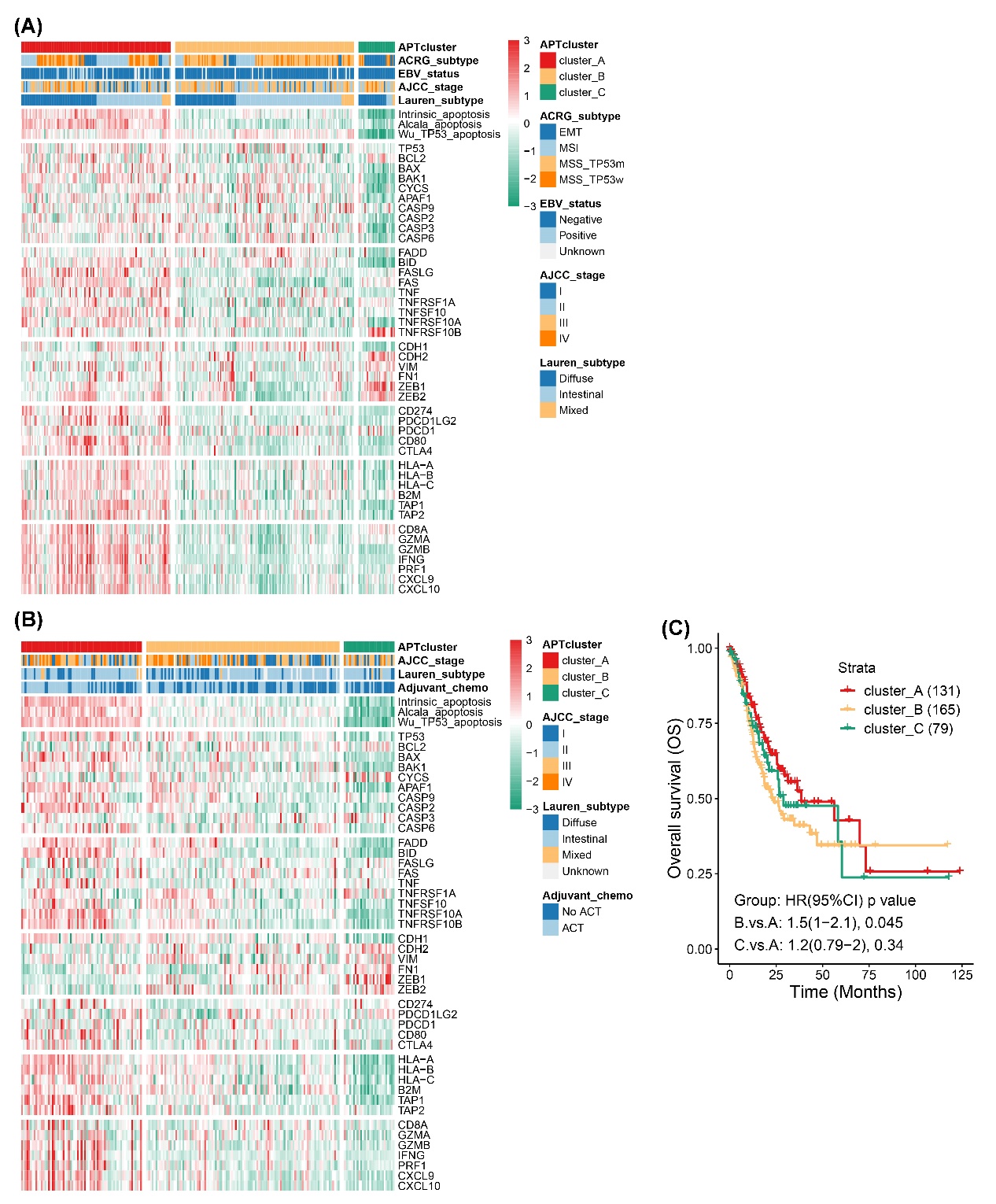
**

**Additional file 5: Figure S1. Identification of three apoptosis signatures for the establishment of an apoptotic phenotype.**

(A) Heatmap showing GSEA results from a panel of public gastric cancer datasets, including the TCGA and several GEO cohorts. All apoptosis signatures were obtained from MSigDB. (B) Cox regression model analysis showing the hazard ratio for overall survival (OS) for each apoptosis signature in the TCGA cohort. (C) Kaplan–Meier survival analysis for patients with high or low ssGSEA scores of each signature in the ACRG cohort.
