# Supplementary material for: CDK5 promotes apoptosis and attenuates chemoresistance in gastric cancer via E2F1 signaling
Source: Cancer Cell Int. 2023 Nov 21;23:286. doi: 10.1186/s12935-023-03112-4 (PMC10664659; doi:10.1186/s12935-023-03112-4)
Supplement: Supplementary file 6 — Additional file 6: Figure S2. Apoptotic phenotype correlates with TCGA subtype and is associated with prognosis in gastric cancer. (A and B) Heatmap showing the expression of marker genes in several functional gene sets in the ACRG (A) and GSE26942 (B) cohorts. (C) Kaplan–Meier survival analysis for patients with different apoptotic phenotypes in the TCGA cohort. [file 12935_2023_3112_MOESM6_ESM.docx]

**
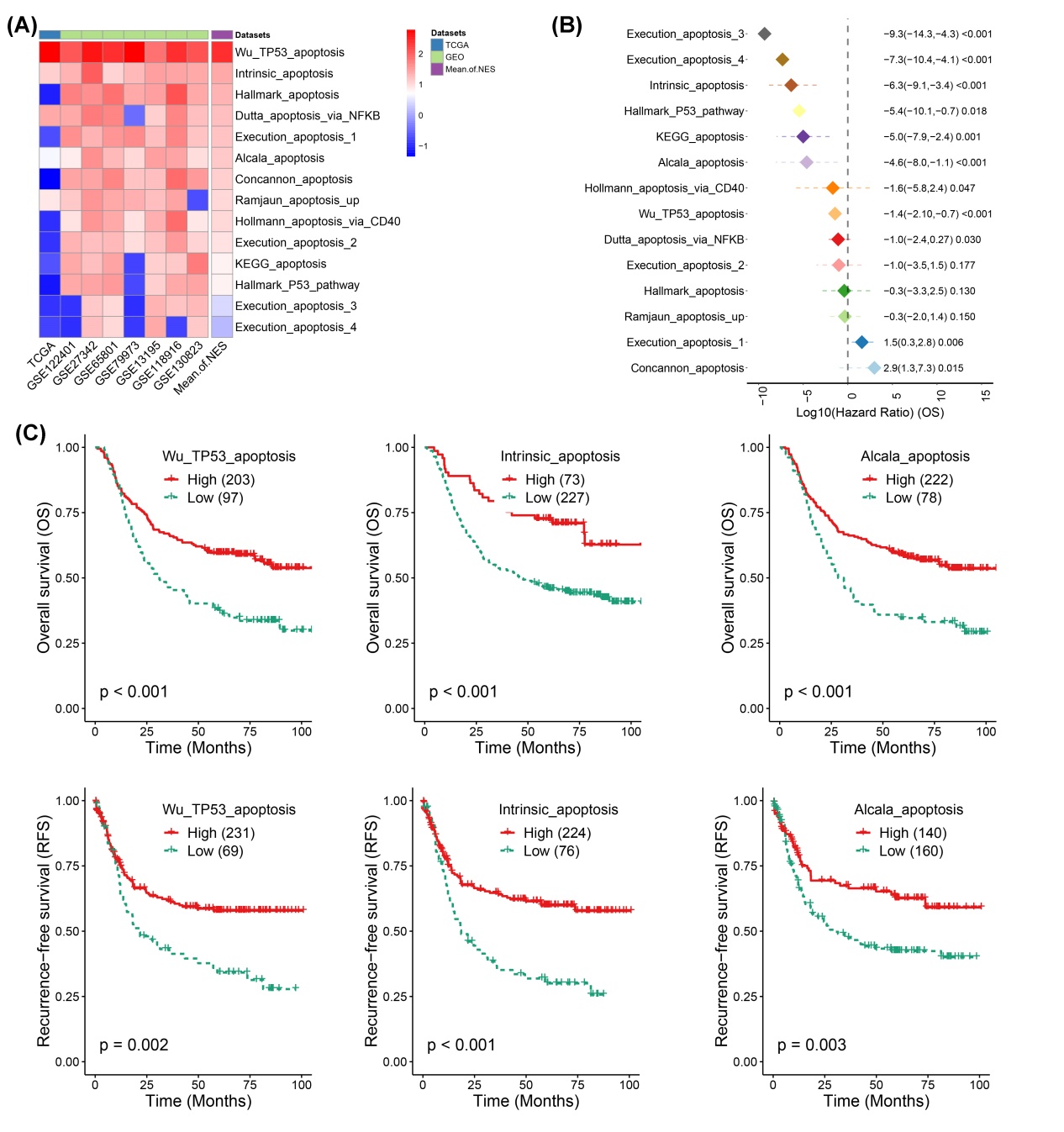
**

**Additional file 6: Figure S2. Apoptotic phenotype correlates with TCGA subtype and is associated with prognosis in gastric cancer**

(A and B) Heatmap showing the expression of marker genes in several functional gene sets in the ACRG (A) and GSE26942 (B) cohorts. (C) Kaplan–Meier survival analysis for patients with different apoptotic phenotypes in the TCGA cohort.
