# Supplementary material for: CDK5 promotes apoptosis and attenuates chemoresistance in gastric cancer via E2F1 signaling
Source: Cancer Cell Int. 2023 Nov 21;23:286. doi: 10.1186/s12935-023-03112-4 (PMC10664659; doi:10.1186/s12935-023-03112-4)
Supplement: Supplementary file 7 — Additional file 7: Figure S3. Proapoptotic tumors are enriched in DNA-repair and immune-inflammatory signaling pathways and predict chemotherapeutic benefits. (A) GSEA was performed in the TCGA cohort using a panel of functional gene sets related to GO biological processes and hallmark items from MSigDB. The values indicate NESs. (B) Frame shift mutation load and frame insertion mutation load were examined among the apoptotic phenotypes in the TCGA cohort. The values were log2 transformed. (C) Somatic mutation and copy number variations were examined for apoptosis signaling, receptor tyrosine kinase signaling, immune checkpoints, and EMT signaling. [file 12935_2023_3112_MOESM7_ESM.docx]

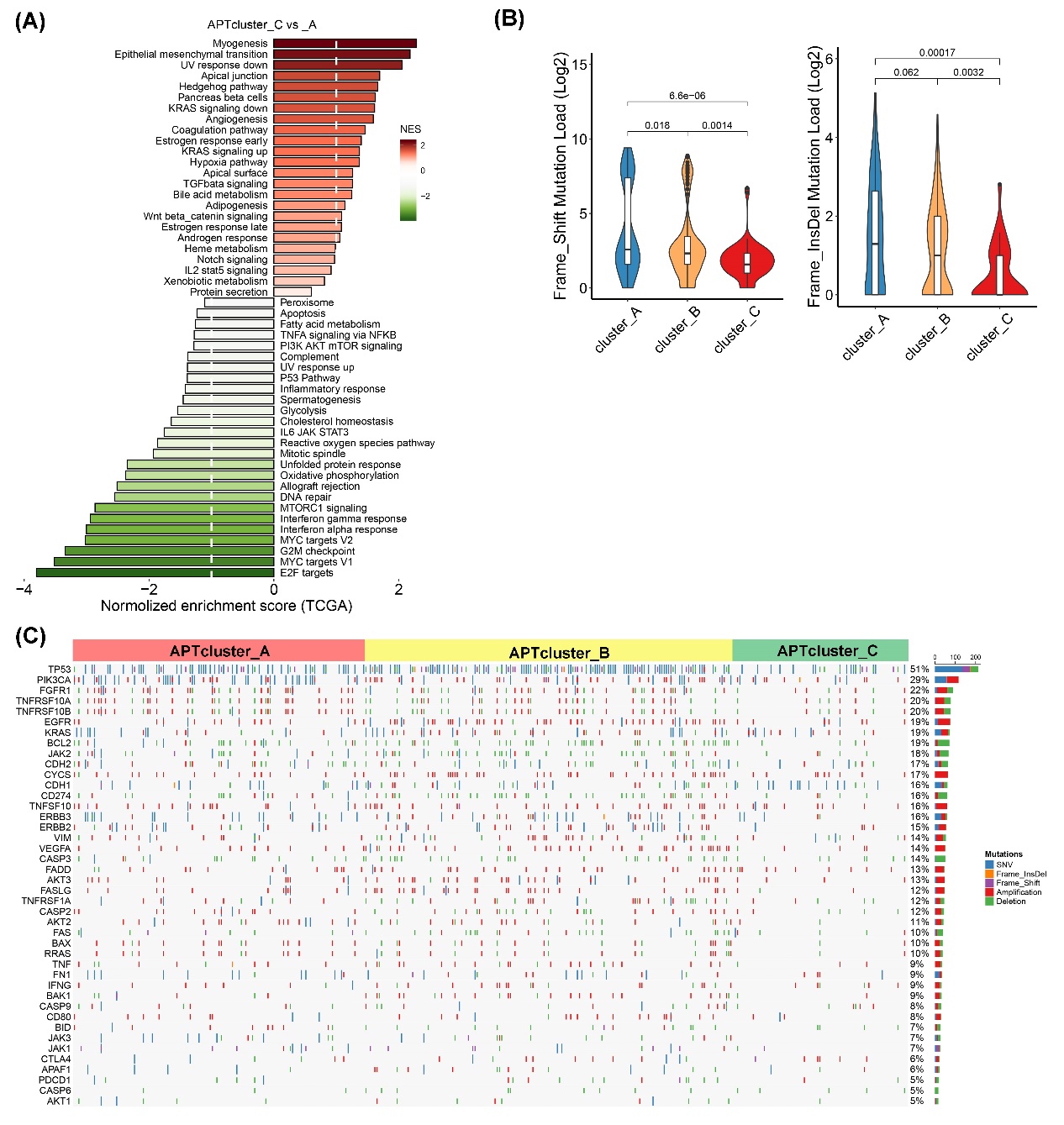


**Additional file 7: Figure S3. Proapoptotic tumors are enriched in DNA-repair and immune-inflammatory signaling pathways and predict chemotherapeutic benefits**

(A) GSEA was performed in the TCGA cohort using a panel of functional gene sets related to GO biological processes and hallmark items from MSigDB. The values indicate NESs. (B) Frame shift mutation load and frame insertion mutation load were examined among the apoptotic phenotypes in the TCGA cohort. The values were log2 transformed. (C) Somatic mutation and copy number variations were examined for apoptosis signaling, receptor tyrosine kinase signaling, immune checkpoints, and EMT signaling.
