# Supplementary material for: CDK5 promotes apoptosis and attenuates chemoresistance in gastric cancer via E2F1 signaling
Source: Cancer Cell Int. 2023 Nov 21;23:286. doi: 10.1186/s12935-023-03112-4 (PMC10664659; doi:10.1186/s12935-023-03112-4)
Supplement: Supplementary file 8 — Additional file 8: Figure S4. CDK5 is a potential proapoptotic gene in gastric cancer. (A and B) DEG analysis of the apoptotic phenotypes was performed in the TCGA, ACRG, and GSE26942 cohorts. Panel A shows 166, 1842, and 429 overlapping DEGs in the antiapoptotic phenotype, and panel B shows 170, 1942, and 351 overlapping DEGs in the proapoptotic phenotype. (C) Copy number variations of CDK5 were examined for each phenotype. (D) Promoter methylation of CDK5 was examined in each phenotype. [file 12935_2023_3112_MOESM8_ESM.docx]

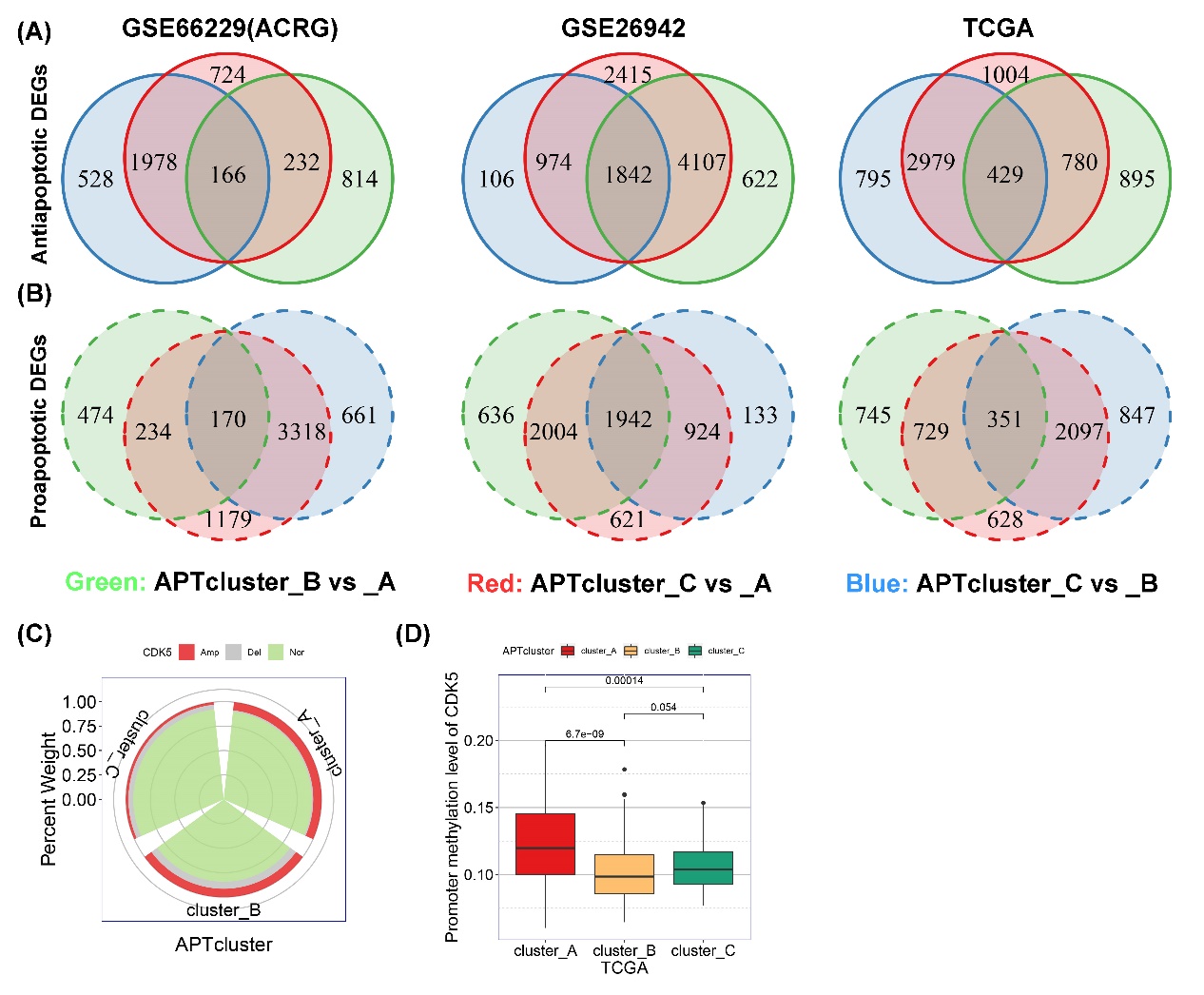


**Additional file 8: Figure S4. *CDK5* is a potential proapoptotic gene in gastric cancer**

(A and B) DEG analysis of the apoptotic phenotypes was performed in the TCGA, ACRG, and GSE26942 cohorts. Panel A shows 166, 1842, and 429 overlapping DEGs in the antiapoptotic phenotype, and panel B shows 170, 1942, and 351 overlapping DEGs in the proapoptotic phenotype. (C) Copy number variations of CDK5 were examined for each phenotype. (D) Promoter methylation of CDK5 was examined in each phenotype.
