# Supplementary material for: CDK5 promotes apoptosis and attenuates chemoresistance in gastric cancer via E2F1 signaling
Source: Cancer Cell Int. 2023 Nov 21;23:286. doi: 10.1186/s12935-023-03112-4 (PMC10664659; doi:10.1186/s12935-023-03112-4)
Supplement: Supplementary file 9 — Additional file 9: Figure S5. CDK5 promotes apoptosis in gastric cancer. (A) Staining (scale bars = 50 μm) for apoptosis assessed caspase 3 activities in the four gastric cancer cell lines after overexpression of CDK5. Green staining indicates caspase 3 activity. (B–D) Western blotting of CDK5, total/cleaved PARP, and total/cleaved caspase 3 in HGC-27 and MKN1 cells. Panel B shows that cells were transfected with the indicated amounts (0.25, 0.5 or 1.0 µg) of pLV-CDK5 expression plasmid (oeCDK5) or empty vector (CTRL). Panel C shows that cells were treated with the indicated amounts (0.25, 0.5 or 1.0 µM) of oxaliplatin (OXA) or DMSO (CTRL). Panel D shows that cells were treated with 0.5 µM OXA or siRNA targeting CDK5. [file 12935_2023_3112_MOESM9_ESM.docx]

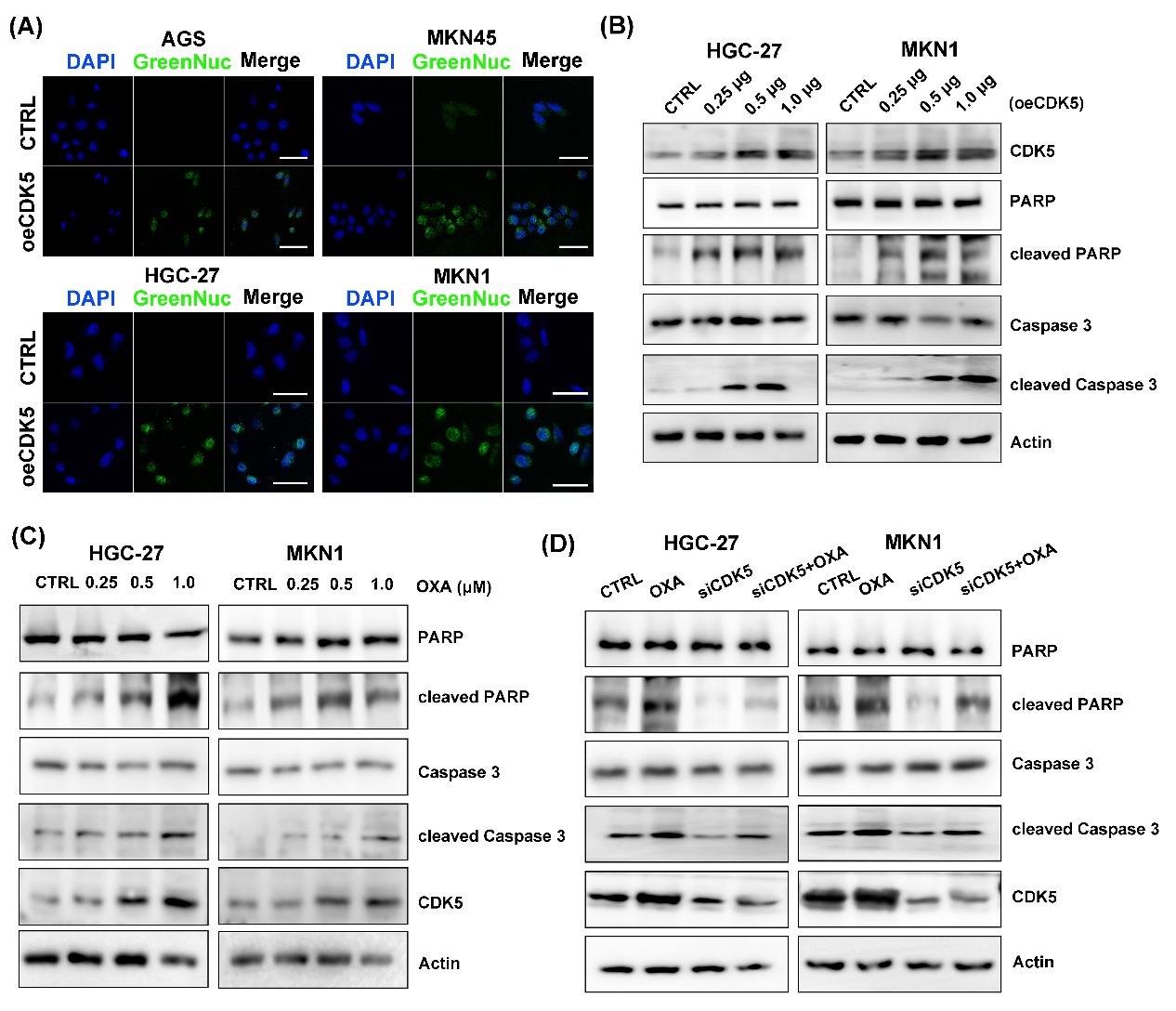


**Additional file 9: Figure S5. CDK5 promotes apoptosis in gastric cancer**

(A) Staining (scale bars = 50 μm) for apoptosis assessed caspase 3 activities in the four gastric cancer cell lines after overexpression of CDK5. Green staining indicates caspase 3 activity. (B–D) Western blotting of CDK5, total/cleaved PARP, and total/cleaved caspase 3 in HGC-27 and MKN1 cells. Panel B shows that cells were transfected with the indicated amounts (0.25, 0.5 or 1.0 μg) of pLV-CDK5 expression plasmid (oeCDK5) or empty vector (CTRL). Panel C shows that cells were treated with the indicated amounts (0.25, 0.5 or 1.0 μM) of oxaliplatin (OXA) or DMSO (CTRL). Panel D shows that cells were treated with 0.5 μM OXA or siRNA targeting CDK5.
