# Supplementary material for: CDK5 promotes apoptosis and attenuates chemoresistance in gastric cancer via E2F1 signaling
Source: Cancer Cell Int. 2023 Nov 21;23:286. doi: 10.1186/s12935-023-03112-4 (PMC10664659; doi:10.1186/s12935-023-03112-4)
Supplement: Supplementary file 10 — Additional file 10: Figure S6. CDK5 induces apoptosis through activation of E2F1 signaling. (A) Western blotting (left) of CDK5, E2F1, DP1, APAF1, and p73 and qRT–PCR analysis (right) of E2F1 and DP1 in HGC-27 and MKN1 cells with CDK5 overexpression or knockdown. (B) Western blots of CDK5, E2F1, total/cleaved PARP, and total/cleaved caspase 3 in HGC-27 and AGS cells transfected with oeCDK5 plasmid and/or siRNA targeting E2F1. (C) Staining (scale bars = 50 μm) for apoptosis examined caspase 3 activity in AGS cells transfected with oeCDK5 plasmid and/or siRNA targeting E2F1. Green staining indicates caspase 3 activity. (D) Cell viability assay evaluated the IC50 curves for oxaliplatin in AGS and HGC-27 cells with CDK5 overexpression and E2F1 silencing. [file 12935_2023_3112_MOESM10_ESM.docx]

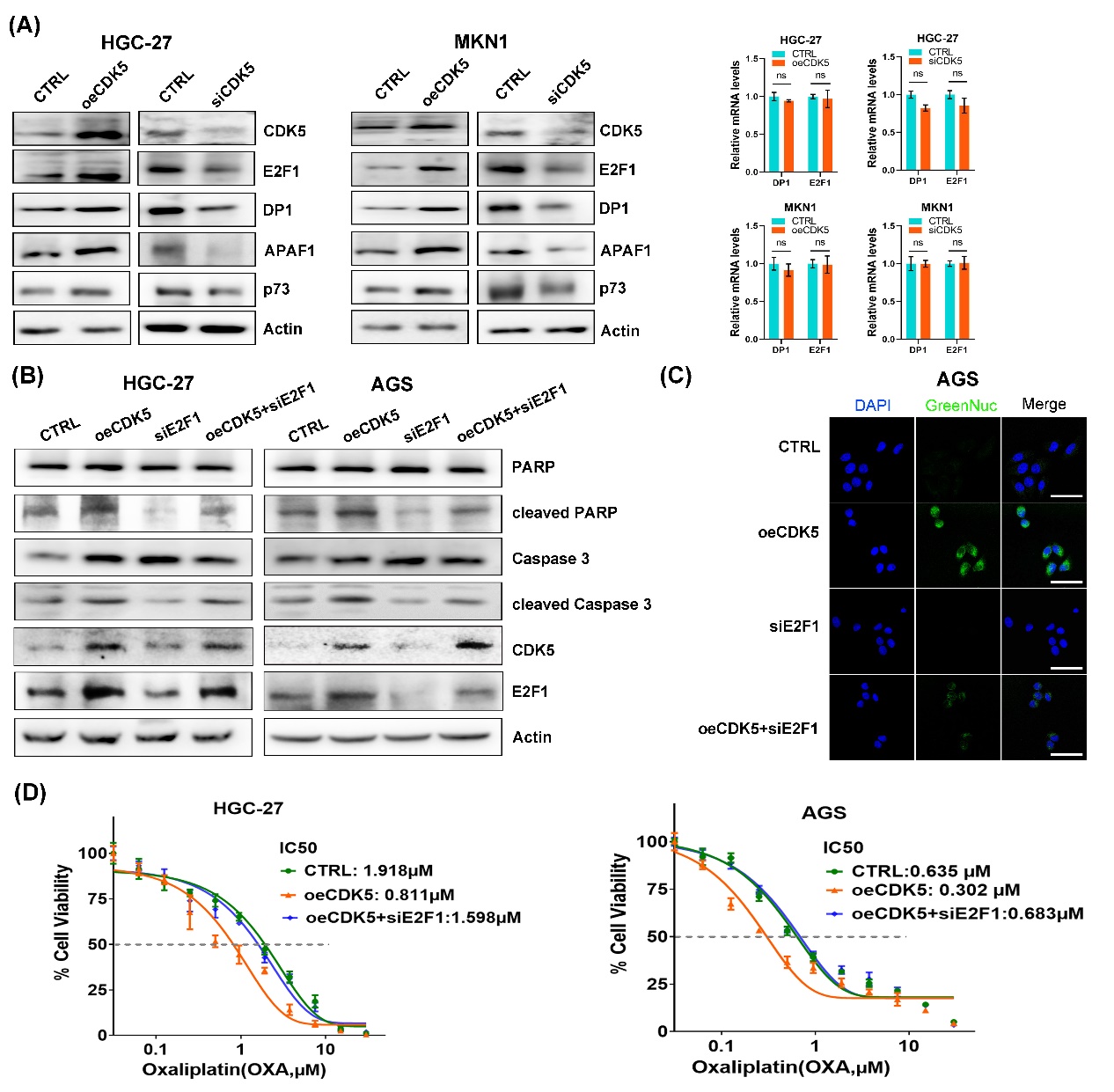


**Additional file 10: Figure S6. CDK5 induces apoptosis through activation of E2F1 signaling**

(A) Western blotting (left) of CDK5, E2F1, DP1, APAF1, and p73 and qRT–PCR analysis (right) of *E2F1* and *DP1* in HGC-27 and MKN1 cells with CDK5 overexpression or knockdown. (B) Western blots of CDK5, E2F1, total/cleaved PARP, and total/cleaved caspase 3 in HGC-27 and AGS cells transfected with oeCDK5 plasmid and/or siRNA targeting E2F1. (C) Staining (scale bars = 50 μm) for apoptosis examined caspase 3 activity in AGS cells transfected with oeCDK5 plasmid and/or siRNA targeting E2F1. Green staining indicates caspase 3 activity. (D) Cell viability assay evaluated the IC50 curves for oxaliplatin in AGS and HGC-27 cells with CDK5 overexpression and E2F1 silencing.
